# Association between the atherogenic index of plasma and metabolic-associated/nonalcoholic fatty liver disease: a systematic review and meta-analysis

**DOI:** 10.3389/fendo.2026.1800577

**Published:** 2026-03-31

**Authors:** Yanxin Zhao, Minshan Huang, Qianlang Ren, Guoqing Wang, Lihong Lu, Dejia Xiong, Xianmei Wang, Lanqing Ma

**Affiliations:** Yunnan Institute of Digestive Disease, The First Affiliated Hospital of Kunming Medical University, Kunming, Yunnan, China

**Keywords:** atherogenic index of plasma (AIP), dyslipidemia, meta-analysis, metabolic dysfunction–associated fatty liver disease (MAFLD), nonalcoholicfatty liver disease (NAFLD)

## Abstract

**Background:**

MAFLD—previously termed NAFLD—has emerged as the most common condition on a global scale. Obesity, insulin resistance, dyslipidemia, and atherosclerosis are closely linked to its pathogenesis. Lipid dysregulation, insulin resistance, and inflammatory status are reflected by the atherogenic index of plasma (AIP), computed as the logarithmic ratio of triglycerides to HDL-C. Although multiple studies have suggested an association between AIP and MAFLD/NAFLD, the reported findings remain inconsistent. The present investigation sought to synthesize available evidence regarding the AIP–MAFLD/NAFLD relationship and to appraise the diagnostic accuracy of AIP.

**Methods:**

This systematic review and meta-analysis was conducted in accordance with the PRISMA 2020 statement. PubMed, Embase, the Cochrane Library, and Web of Science were searched through October 2025. Fixed- or random-effects models were applied according to heterogeneity. Weighted mean difference (WMD) and pooled odds ratios (ORs) were calculated. Diagnostic performance was evaluated using a bivariate random-effects model to obtain pooled sensitivity, specificity, and summary receiver operating characteristic (SROC) curves. Subgroup analyses were performed by diagnostic criteria, age, diabetes status, BMI, and region. Meta-regression explored the influence of demographic and metabolic covariates. Sensitivity analyses and publication bias assessment using Egger regression and Deeks’ asymmetry test were also conducted.

**Results:**

Twenty observational studies involving 245,571 adults were included. AIP levels were significantly higher in MAFLD/NAFLD patients than in controls (WMD = 0.26; 95% CI: 0.21–0.30). Elevated AIP was significantly associated with MAFLD/NAFLD risk (pooled odds ratio = 3.18; 95% CI: 2.54–3.98). Subgroup analyses demonstrated greater consistency in studies using MAFLD diagnostic criteria and among obese populations. Diagnostic meta-analysis using a bivariate random-effects model yielded a pooled sensitivity of 0.73 and specificity of 0.65, with a summary AUC of 0.75 (95% CI: 0.71–0.79), indicating moderate diagnostic accuracy. Sensitivity analyses confirmed robustness, and no significant publication bias was detected.

**Conclusions:**

Elevated AIP is significantly associated with increased MAFLD/NAFLD risk and shows moderate diagnostic performance. As a simple and cost-effective index derived from routine lipid profiles, AIP may serve as a useful early screening tool for individuals at elevated risk of fatty liver disease and cardiometabolic comorbidities. Further prospective studies are warranted to clarify causality and clinical utility.

**Systematic review registration:**

https://www.crd.york.ac.uk/PROSPERO/home, identifier CRD42025116919.

## 1 Background

MAFLD, formerly designated NAFLD, now ranks as the most prevalent chronic hepatic condition globally, affecting approximately one-third of the adult population (1). The increasing prevalence of fatty liver disease among young adults, middle-aged individuals, and metabolically high-risk populations poses a major public health challenge, driven by globalization, urbanization, Westernized dietary patterns, and sedentary lifestyles (2, 3). The MAFLD construct underscores metabolic dysfunction as a core pathogenic driver, in contrast to NAFLD, which relies on a diagnosis-of-exclusion approach. Despite differences in nomenclature and diagnostic criteria, the two entities show substantial overlap in pathological basis, clinical manifestations, and disease outcomes, representing the same spectrum of metabolically related liver disease (4). MAFLD extends beyond liver pathology, showing strong associations with obesity, type 2 diabetes mellitus, dyslipidemia, and atherosclerotic cardiovascular disease (5, 6). Early detection of at-risk individuals with metabolic and cardiovascular abnormalities is thus pivotal to preventing disease advancement and mitigating future complication burden.

AIP, calculated as log(TG/HDL-C), serves as a composite indicator integrating atherogenic lipid patterns, small dense LDL phenotype, insulin resistance, and systemic inflammation (7–9). The accessibility, cost-effectiveness, and sensitivity of AIP have positioned it as an attractive cardiovascular risk marker, attracting increasing attention in metabolic disease investigations (7–12). Given the strong interconnection between fatty liver disease, metabolic abnormalities, and atherosclerotic risk, AIP holds potential as a predictor of fatty liver disease. However, the available evidence remains inconsistent. While several studies have reported significant AIP-NAFLD/MAFLD associations (13–31), others have shown substantial effect size attenuation following adjustment for BMI, insulin resistance, and other metabolic or cardiovascular risk factors in multivariable models (27, 32). Population characteristics, metabolic comorbidity spectrum, and covariate selection may influence this association, as suggested by these findings. In addition, marked heterogeneity in study design, disease definition, and study populations across previous investigations further limits the comparability and robustness of existing conclusions.

Although a meta-analysis published in 2022 examined the association between AIP and NAFLD, that study included only six articles, with limited outcome measures, and the sample size and scope were insufficient to support robust conclusions (33). Given the substantial number of new studies published recently, an updated and more comprehensive systematic review and meta-analysis is warranted. By synthesizing data from both NAFLD and MAFLD studies, the current analysis aims to quantify AIP association magnitude and diagnostic performance across varying diagnostic criteria. This study was designed to bridge knowledge gaps and provide an evidence-based rationale for AIP application in fatty liver disease risk stratification, early detection, and cardiometabolic care.

## 2 Methods

PRISMA 2020 guidelines governed all aspects of study conduct and reporting (34). Protocol registration was completed via the International Prospective Register of Systematic Reviews (CRD420251169195).

### 2.1 Data sources

Comprehensive literature searches spanning database inception to October 12, 2025, were executed across PubMed, Embase, the Cochrane Library, and Web of Science. No language restrictions were imposed to ensure comprehensive literature coverage.

The search strategy integrated Medical Subject Headings with free-text terminology. Fatty liver–related terms, including “non-alcoholic fatty liver disease”/”NAFLD,” “metabolic dysfunction-associated fatty liver disease”/”MAFLD,” “fatty liver,” and “hepatic steatosis,” together with AIP-related terms, including “atherogenic index of plasma” and “AIP,” were used as core keywords. Logical combinations were applied across databases, for example: (NAFLD OR MAFLD OR “fatty liver” OR “hepatic steatosis”) AND (“atherogenic index of plasma” OR AIP), with appropriate adjustments made according to the specific characteristics of each database. Manual screening of reference lists from included articles was conducted to ensure comprehensive literature capture (**Supplementary Table S1**).

### 2.2 Study selection

EndNote 20 was used for managing retrieved records and removing duplicates. Two investigators (Zhao and Huang) independently screened titles and abstracts before proceeding to full-text evaluation of candidate studies. Disagreements were resolved through discussion, with a third investigator consulted when necessary to reach consensus.

The PECOS framework guided development of eligibility criteria. Adults aged ≥18 years with NAFLD or MAFLD diagnosed via imaging (ultrasonography, computed tomography, or magnetic resonance imaging), serum-based scores (e.g., fatty liver index), or liver histology were included, along with studies identifying NAFLD/MAFLD through screening. The exposure of interest was AIP, defined as log(TG/HDL-C), reported either as a continuous variable, per unit or per standard deviation increase, or as a categorical variable, such as high versus low groups or quantiles. The comparison group comprised individuals with lower AIP levels or those without NAFLD or MAFLD. Outcomes encompassed AIP-NAFLD/MAFLD association measures (prevalence or incidence), effect estimates (odds ratios or hazard ratios), and diagnostic performance (AUC). Cross-sectional, case–control, and cohort designs were eligible for inclusion.

Exclusion criteria included non-original research (reviews, editorials, case reports, conference abstracts, or letters), animal studies, and absence of relevant outcomes. When multiple studies originated from the same study population or database, only one study was selected to avoid data duplication. In such cases, prespecified selection criteria were applied to determine the most appropriate study. Priority was given to the following factors: (1) the study with the largest sample size; (2) the study with the most comprehensive multivariable-adjusted model; (3) the study with the longest follow-up duration in cohort studies; and (4) the study with the most complete reporting of outcome measures (e.g., OR, WMD, or AUC). This approach ensured that each population was included only once in the meta-analysis while retaining the most informative data.

### 2.3 Risk of bias assessment

Two reviewers (Zhao and Huang) independently evaluated methodological quality using National Institutes of Health tools designed for observational research. T Design-specific instrument versions were selected: one for cross-sectional and cohort studies, another for case–control studies. Assessment discrepancies were resolved via discussion, with a third investigator adjudicating when necessary.

Multiple domains are assessed by the National Institutes of Health tool: research question clarity, study population definition, sample source, participation rate, sample size justification, statistical power, exposure and outcome measurement accuracy, temporal relationships, follow-up adequacy, and confounder control. The tool comprises 14 items for cross-sectional and cohort studies and 12 items for case–control studies. National Institutes of Health criteria guided quality categorization: high (low bias risk), moderate (moderate bias risk), and low (high bias risk).

### 2.4 Data extraction

Data were independently abstracted by two reviewers (Zhao and Huang) onto a standardized extraction form and subsequently cross-checked for accuracy. A third investigator reviewed and resolved any discrepancies. Corresponding authors were contacted to obtain additional information when original reports contained missing or unclear data. All extracted data were re-verified before quantitative synthesis.

Data extraction covered study-level information (author, year, country/region, design, population), participant demographics (sample size, sex, age, BMI, waist circumference, smoking), metabolic parameters (blood pressure, glycemia, lipids, hepatic enzymes, uric acid), and outcome measures (AIP values, effect estimates, and diagnostic accuracy data including sensitivity, specificity, and when available, true/false positives and negatives). When multiple models were reported within the same study, the model with the most comprehensive adjustment for confounders was preferentially extracted.

### 2.5 Data synthesis

STATA version 15.0 was used for all statistical analyses. Meta-analysis was conducted when at least two studies reported comparable effect estimates; otherwise, a qualitative synthesis was provided. Pooled effect measures included odds ratios, hazard ratios, weighted mean difference (WMD) for AIP levels. Heterogeneity was quantified via the Q test and I^2^ statistic. A random-effects model was selected for analyses with I^2^ exceeding 50% or heterogeneity P below 0.05; otherwise, a fixed-effect model was applied. Stratified analyses were undertaken according to diagnostic criteria (NAFLD/MAFLD), age, diabetes mellitus status, BMI, and geographic region. Meta-regression analyses at the study level examined potential moderators including mean age, proportion of female participants, BMI, smoking prevalence, systolic and diastolic blood pressure, waist circumference, fasting glucose, ALT, AST, and uric acid. Pooled estimate stability was tested by sequentially omitting each study. Egger regression was used to assess publication bias, with trim-and-fill adjustment applied when substantial bias was detected.

For diagnostic accuracy analysis, studies reporting sensitivity and specificity data were included. Pooled sensitivity and specificity were estimated using a bivariate random-effects model, and a summary receiver operating characteristic (SROC) curve was constructed. The threshold effect was assessed by examining the correlation between logit-transformed sensitivity and the false-positive rate using Spearman correlation analysis. Publication bias was evaluated using the Deeks funnel plot asymmetry test.

## 3 Results

### 3.1 Literature search process

**Figure 1:**
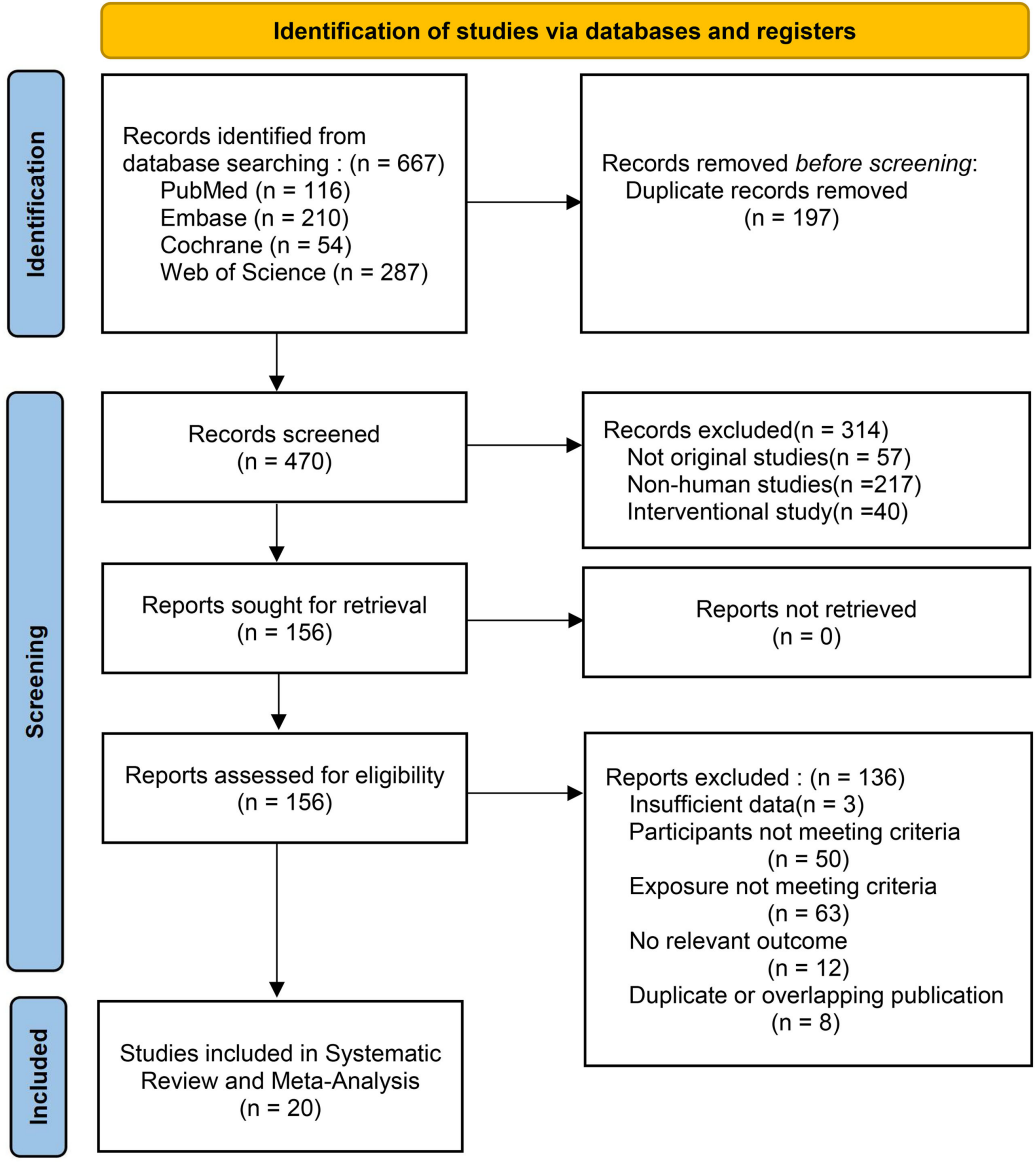
The PRISMA flow diagram of the identification, screening, and inclusion methodology of the studies.

Database searching identified 667 records. Following exclusion of 197 duplicates, 470 articles underwent title and abstract screening. During this initial screening stage, 314 articles were excluded. Full-text assessment was subsequently performed for 156 articles, of which 136 were excluded for the following reasons: insufficient data to extract AIP values or effect estimates (n = 3); study populations consisting of children or other special populations not meeting the predefined criteria (n = 50); inappropriate exposure definition, lack of explicit inclusion of AIP as the exposure of interest, or unclear exposure definition (n = 63); absence of NAFLD or MAFLD outcome reporting (n = 12); and duplicate or overlapping publications derived from the same cohort or database (n = 8). A total of 20 studies fulfilled eligibility criteria and were included (**Figure 1**) (13–32).

### 3.2 Characteristics of the included studies

The analysis incorporated 20 observational studies spanning 2018 to 2025, with a combined sample of 245,571 participants. Study designs included 18 cross-sectional studies, one case-control study, and one cohort study. Study populations were drawn from China, Korea, the United States, Italy, Iran, Pakistan, Romania, Türkiye, and Saudi Arabia. While most studies enrolled general adult populations, some specifically targeted individuals with type 2 diabetes mellitus, obesity, or pregnancy.

Participant characteristics included: mean age 32.07–58.45 years, BMI 22.64–31.32 kg/m^2^, and female proportion 27.78%–100%. Diagnosis of NAFLD or MAFLD was primarily based on imaging modalities, with a small number of studies using serum-based scores, such as the fatty liver index (23). Eleven studies applied NAFLD diagnostic criteria, eight applied MAFLD diagnostic criteria, and one used the broader term “fatty liver.” All studies considered the AIP as the primary exposure. Of these, 11 studies reported odds ratios or hazard ratios for AIP-NAFLD/MAFLD association, 16 compared AIP levels between groups, and 13 reported AUC values for diagnostic evaluation (**Table 1**).

**Table 1:** Main characteristics of studies in this meta-analysis.

Author (Year)	Country	Study design	Sample size (n)	Mean age (years)	Female(%)	BMI (kg/m^2^)	SBP/DBP (mmHg)	Diagnostic criteria (NAFLD/MAFLD/fatty liver)	Population type	Outcome measure
Xie 2019 (13)	China	Cross-sectional study	7,838	43.98	43	24.02	123.69/74.71	Fatty Liver	General adults	AIP, AUC
Nida 2025 (14)	Pakistan	Cross-sectional study	502	44.4	39.84	26.78	126.91/74.1	MAFLD	General adults	AIP
Song 2025 (15)	China	Cohort study	25,366	40	54.06	23.14	124.38/74.8	MAFLD	General adults	AIP, HR, AUC
Duan 2022 (16)	China	Cross-sectional study	864	39.81	27.78	26.56	130.46/80.73	MAFLD	General adults	AIP, OR, AUC
Matteis 2025 (17)	Italy	Cross-sectional study	1,496	57.2	50.97	NR	NR	MAFLD	General adults	OR
Samimi 2022 (18)	Iran	Case–control study	2,547	58.45	55.63	29.26	133.37/78.63	MAFLD	Type 2 diabetes patients	AIP, OR, AUC
Ciftel 2024 (19)	Turkey	Cross-sectional study	695	38.85	63.31	31.32	NR	MAFLD	General adults	AIP, AUC
Chen 2024 (20)	US	Cross-sectional study	1,090	44	49.06	27.8	NR	MAFLD	General adults	OR, AUC
Efrem 2022 (21)	Romania	Cross-sectional study	200	56.55	51.55	NR	NR	MAFLD	Type 2 diabetes patients	AIP
Xie 2021 (22)	China	Cross-sectional study	1,748	43.9	34.04	24.02	123/75.11	NAFLD	General adults	AIP, OR, AUC
Lin 2022 (23)	China	Cross-sectional study	1,074	56.18	49.53	NR	137.01/79.52	NAFLD	Type 2 diabetes patients	AIP, AUC
Peng 2023 (24)	China	Cross-sectional study	2,318	43	45.77	24.7	118/78	NAFLD	General adults	OR, AUC
Ruan 2024 (25)	China	Cross-sectional study	245	53.16	42.86	22.37	120.74/73	NAFLD	General adults	AIP
Mohammedsaeed 2025 (26)	Saudi Arabia	Cross-sectional study	1,500	44.83	51.33	22.48	NR	NAFLD	General adults	AIP
Dong 2020 (32)	China	Cross-sectional study	78,304	44.61	42.41	25.48	NR	NAFLD	General adults	AIP, AUC
Shuai 2025 (27)	Korea	Cross-sectional study	586	32.07	100	21.64	NR	NAFLD	Pregnant women	AIP, OR
Fadaei 2019 (28)	Iran	Cross-sectional study	82	50.6	NR	NR	128.9/80.28	NAFLD	General adults	AIP
Wang 2025 (29)	China	Cross-sectional study	6,378	57.8	36.05	26.79	133/80	NAFLD	Type 2 diabetes patients	AIP, OR, AUC
Wang 2018 (30)	China	Cross-sectional study	538	42.19	47.77	24.34	NR	NAFLD	Patients with obesity	OR, AUC
Liu 2022 (31)	China	Cross-sectional study	112,200	42.31	47.8	30.69	124.71/74.64	NAFLD	General adults	AIP, OR, AUC

BMI, body mass index; SBP, systolic blood pressure; DBP, diastolic blood pressure; NAFLD, non-alcoholic fatty liver disease; MAFLD, metabolic dysfunction-associated fatty liver disease; AIP, atherogenic index of plasma; AUC, area under the receiver operating characteristic curve; OR, odds ratio; RR, risk ratio; NR, not reported.

### 3.3 Quality assessment of the included studies

Risk of bias was assessed for all included studies using National Institutes of Health quality assessment tools. High quality ratings were assigned to nine studies, while 11 studies received moderate quality ratings. No studies were classified as low quality. All studies clearly defined their research objectives and study populations, and exposure and outcome measures were well specified and reliably assessed. However, most cross-sectional studies did not report sample size calculations or statistical power justification and generally lacked random sampling and blinded assessment, which may introduce selection bias and information bias. The cohort study demonstrated better performance with respect to exposure temporality and follow-up completeness, although residual confounding could not be entirely excluded. Included studies generally demonstrated moderate-to-high methodological quality, yielding a reasonably robust evidence foundation for quantitative synthesis (**Supplementary Table 2**).

### 3.4 Meta-analysis results

#### 3.4.1 Differences in AIP levels between NAFLD/MAFLD and control groups

**Figure 2:**
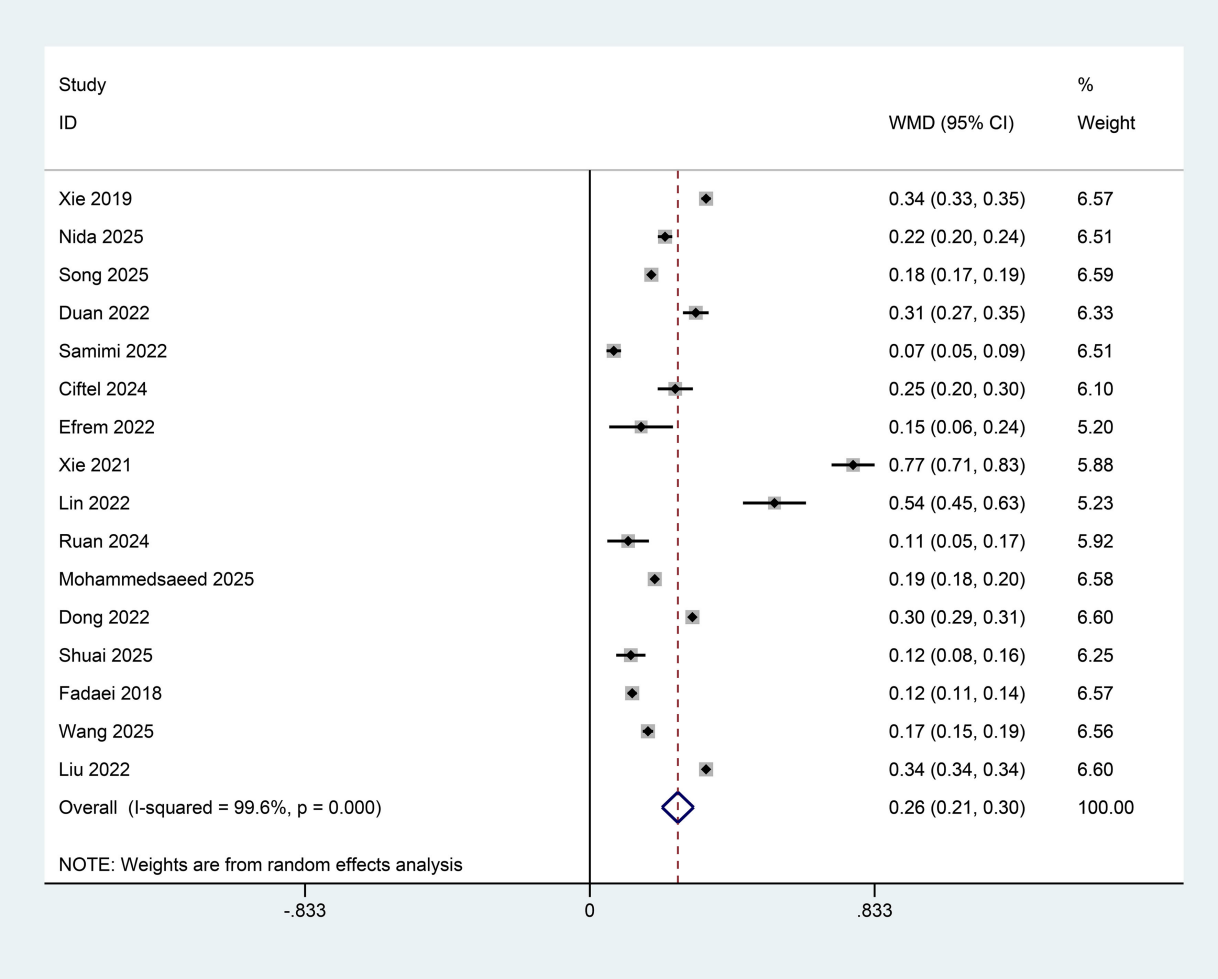
Forest plot showing the difference in AIP levels between NAFLD/MAFLD patients and control groups (weighted mean difference, 95% CI).

Sixteen studies compared AIP levels between participants with NAFLD or MAFLD and controls. Given substantial heterogeneity across studies (I² = 99.6%, P < 0.001), a random-effects model was applied. The pooled WMD was 0.26 (95% confidence interval: 0.21–0.30), indicating that AIP levels were significantly higher in participants with NAFLD or MAFLD than in controls (**Figure 2**).

#### 3.4.2 Association between AIP and risk of NAFLD/MAFLD

**Figure 3:**
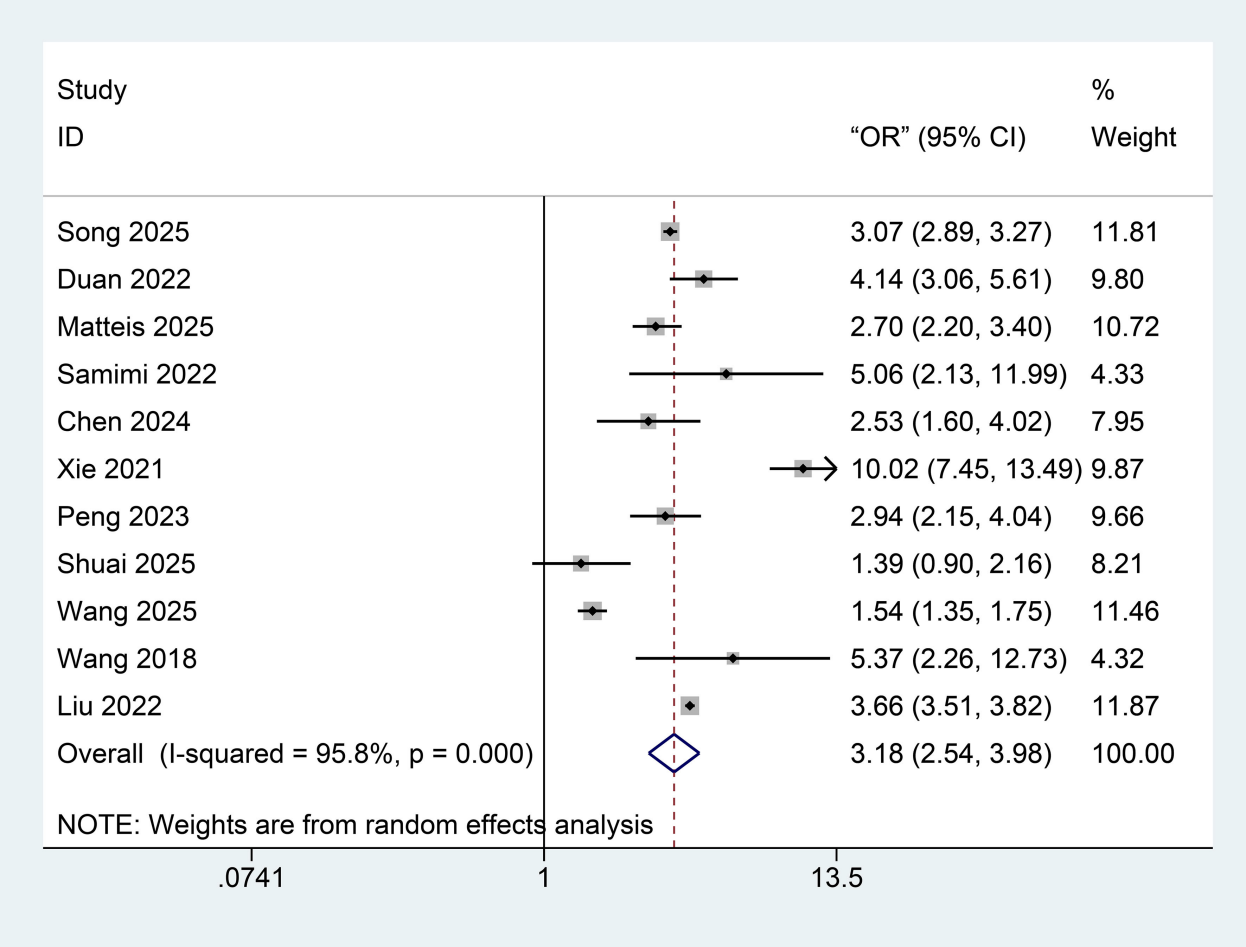
Forest plot showing the association between AIP and risk of NAFLD/MAFLD (odds ratio, 95% CI).

AIP–NAFLD/MAFLD risk associations were examined across 11 studies. Substantial heterogeneity (I^2^ = 95.8%, P < 0.001) necessitated random-effects modeling. Increased AIP was significantly linked to heightened NAFLD/MAFLD risk, with a pooled odds ratio of 3.18 (95% confidence interval: 2.54–3.98) (**Figure 3**).

#### 3.4.3 Diagnostic performance of AIP for NAFLD/MAFLD

**Figure 4:**
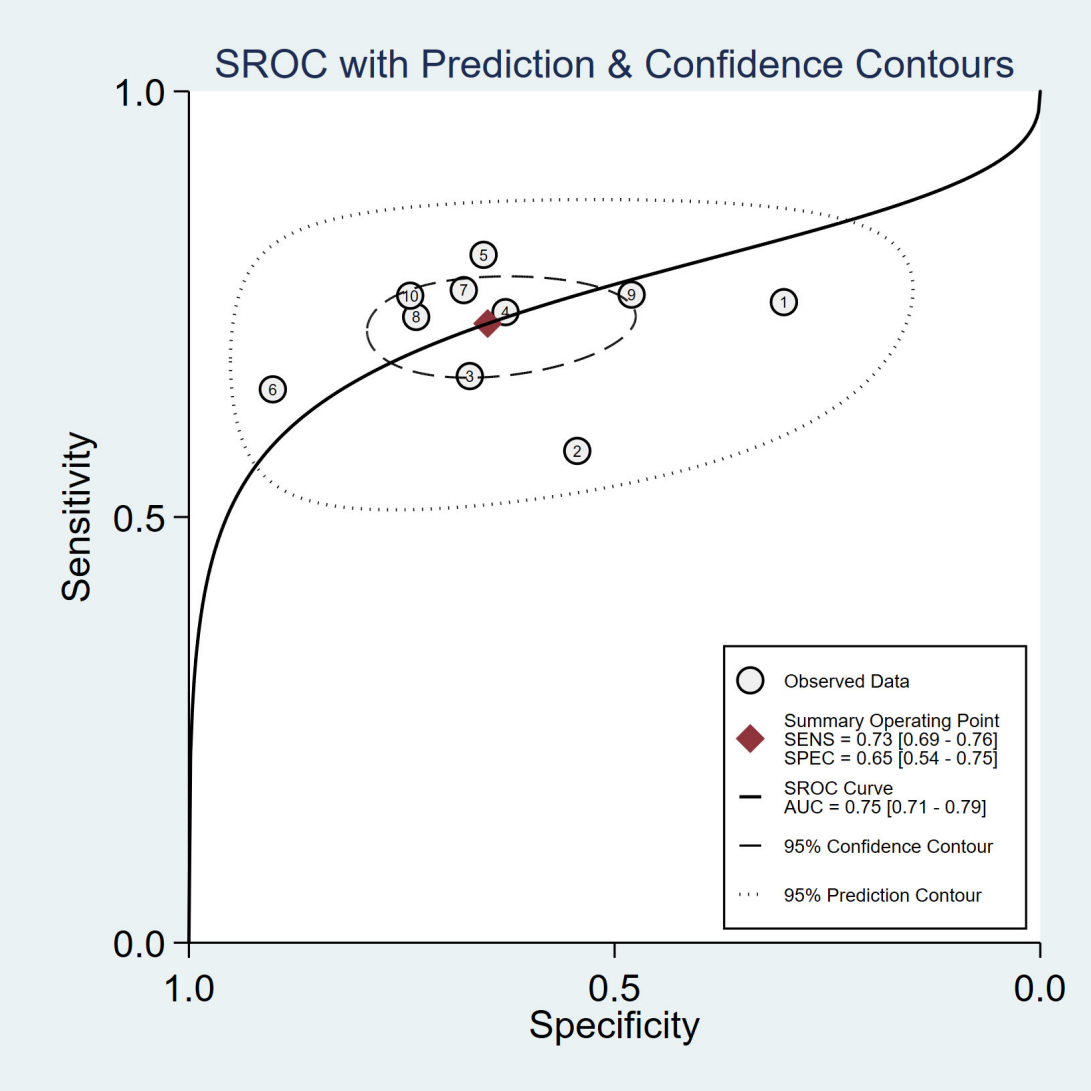
Summary receiver operating characteristic (SROC) curve of AIP for the diagnosis of NAFLD/MAFLD using a bivariate random-effects model.

A total of 13 studies evaluated the diagnostic performance of AIP for NAFLD/MAFLD, of which 10 studies provided complete sensitivity and specificity data (18, 20–22, 24–26, 29, 32, 34) and were included in the bivariate random-effects model analysis. Spearman correlation analysis indicated no significant threshold effect (P = 0.701). The pooled sensitivity was 0.73 (95% CI: 0.69–0.76), the pooled specificity was 0.65 (95% CI: 0.54–0.75), and the summary AUC under the SROC curve was 0.75 (95% CI: 0.71–0.79), indicating moderate discriminatory ability of AIP. Given the limited number of studies included in the diagnostic accuracy analysis and the small number of studies within each subgroup after further stratification, subgroup analysis and diagnostic meta-regression were not performed to avoid potential bias arising from model instability (**Figure 4**; **Supplementary Figure S18**).

#### 3.4.4 Subgroup and meta-regression analyses

Heterogeneity sources were investigated via subgroup stratification by diagnostic criteria, age, diabetes status, BMI, and geographic region. Although effect sizes varied slightly across subgroups, the overall direction of the association remained consistent, supporting the robustness and consistency of the findings. The pooled odds ratio in type 2 diabetes mellitus populations was 2.58 (95% CI: 0.81–8.18), failing to reach statistical significance in diabetes-stratified analyses. The limited study count and random-effects modeling, known to generate wider confidence intervals under heterogeneous conditions, may partly explain this observation; therefore, these results should be interpreted with caution. Subgroup analyses of the association between AIP and NAFLD or MAFLD revealed reduced heterogeneity within strata defined by diagnostic criteria and BMI, with higher internal consistency observed in studies using MAFLD definitions and in populations with overweight or obesity, indicating that these factors may contribute to overall heterogeneity (**Table 2**; **Supplementary Figures S1**–**S10**).

**Table 2:** Subgroup analyses of the relationship between AIP and NAFLD/MAFLD based on diagnostic criteria, age, diabetes status, BMI, and study region.

Subgroup	The Difference in AIP levels between groups			The association between AIP and NAFLD/MAFLD		
	Study	WMD [95%CI]	I^2^%	Study	OR [95%CI]	I^2^%
Total	16	0.26 (0.21, 0.30)	99.6	11	3.18 (2.54, 3.98)	95.8
Diagnostic Criteria						
MAFLD	6	0.20 (0.14, 0.25)	97.1	5	3.12 (2.68, 3.62)	43.2
NAFLD	9	0.29 (0.23, 0.35)	99.6	6	3.21 (1.93, 5.33)	97.8
Age (years)						
<45	10	0.30 (0.25, 0.35)	99.6	8	3.57 (2.94, 4.34)	92.4
>45	6	0.18 (0.12, 0.23)	96.2	3	2.43 (1.43, 4.13)	91.8
With Type 2 diabetes						
NO	12	0.27 (0.22, 0.32)	99.6	9	3.44 (2.87, 4.12)	91.8
YES	4	0.22 (0.12, 0.33)	97.7	2	2.58 (0.81, 8.18)	86.0
BMI(kg/m2)						
<25	7	0.28 (0.22, 0.33)	99.7	4	2.67 (1.97, 3.62)	98.1
>25	6	0.19 (0.14, 0.24)	97.8	4	3.77 (2.74, 5.19)	30.8
Area						
China	9	0.33 (0.28, 0.38)	99.6	7	3.59 (2.73, 4.72)	97.2
Others	7	0.16 (0.12, 0.20)	96.6	4	2.43 (1.63, 3.61)	69.8

AIP, atherogenic index of plasma; NAFLD, non-alcoholic fatty liver disease; MAFLD, metabolic dysfunction-associated fatty liver disease; BMI, body mass index; OR, odds ratio; WMD, weighted mean difference.

Meta-regression examined the effects of demographic and metabolic covariates (age, female proportion, BMI, nonsmoker proportion, blood pressure, waist circumference, fasting glucose, alanine aminotransferase, aspartate aminotransferase, and uric acid) on pooled WMD and odds ratio. None of the covariates showed statistically significant associations with the pooled outcomes, with all regression P values exceeding 0.05 (**Table 3**; **Supplementary Figures S11**, **S12**).

**Table 3:** Meta-regression of the association between AIP and NAFLD/MAFLD.

Covariate	Difference in AIP levels between groups (WMD)		The association between AIP and NAFLD/MAFLD (OR)	
	No. of studies	p value	No. of studies	p value
Age, years	16	0.624	11	0.931
Female, %	15	0.158	11	0.119
BMI, kg/m²	13	0.496	8	0.286
Non-smokers, %	4	0.220	5	0.677
SBP, mmHg	11	0.841	7	0.554
DBP, mmHg	11	0.783	7	0.368
WC, cm	8	0.801	6	0.986
FBG, mmol/L	14	0.594	9	0.157
ALT, U/L	14	0.761	8	0.349
AST, U/L	14	0.688	8	0.187
UA, μmol/L	6	0.214	4	0.082

AIP, atherogenic index of plasma; NAFLD, non-alcoholic fatty liver disease; MAFLD, metabolic dysfunction-associated fatty liver disease; OR, odds ratio; WMD, weighted mean difference; BMI, body mass index; SBP, Systolic blood pressure; DBP, Diastolic blood pressure; WC, Waist circumference; FBG, Fasting blood glucose; ALT, Alanine aminotransferase; AST, Aspartate aminotransferase; UA, Uric acid.

#### 3.4.5 Sensitivity analysis and publication bias

Sequential exclusion of individual studies was performed in sensitivity analyses examining AIP level differences, AIP-NAFLD/MAFLD risk association. Pooled effect estimates and 95% confidence intervals remained stable following exclusion of any single study, confirming robustness and absence of undue influence from individual studies. Publication bias for WMD and odds ratio outcomes was assessed using Egger’s regression test, which showed no significant small-study effects (P = 0.133 and P = 0.671, respectively). For diagnostic accuracy analysis, Deeks’ funnel plot asymmetry test was applied, and no significant publication bias was detected (P = 0.062) (**Supplementary Figures 13**-**S17**).

## 4 Discussion

Based on 20 observational studies involving 245,571 adults, this study systematically evaluated the association between the AIP and MAFLD or NAFLD and further examined its diagnostic performance. The main findings demonstrate significant AIP-NAFLD/MAFLD risk associations, markedly elevated AIP values in fatty liver disease patients compared with controls, and moderate AIP diagnostic performance. These findings support the potential utility of this lipid-derived index for detecting individuals at elevated fatty liver disease risk.

These findings align with previous single-center studies and narrative reviews reporting elevated AIP prevalence in fatty liver disease populations and persistent significant associations with NAFLD/MAFLD following multivariable adjustment. The AIP metric encapsulates an atherogenic lipid profile characterized by raised triglyceride and reduced HDL-C concentrations. This metabolic phenotype is closely associated with insulin resistance, increased free fatty acid flux, intrahepatic lipid droplet accumulation, and low-grade chronic inflammation (35–37). Hepatic glucose and fatty acid synthesis are promoted by insulin resistance, while HDL-C dysfunction impairs reverse cholesterol transport, resulting in hepatic lipid accumulation and facilitating progression from simple steatosis to steatohepatitis and fibrosis (37–40). In addition, an elevated AIP state is often accompanied by increased oxidative stress and upregulation of inflammatory mediators, which may accelerate hepatocellular injury and fibrotic remodeling (41). Insulin resistance promotes hepatic glucose and fatty acid synthesis and exacerbates the imbalance between lipid synthesis and oxidation through activation of SREBP-1c, inhibition of AMPK, and disruption of PPAR signaling pathways, while HDL-C dysfunction impairs reverse cholesterol transport, leading to further intrahepatic lipid accumulation and driving the progression from simple steatosis to steatohepatitis and fibrosis (37–40, 42). In addition, a high AIP state is often accompanied by enhanced oxidative stress and aberrant expression of lipid metabolism-related genes, further amplifying inflammatory responses and lipotoxicity (43). Beyond metabolic abnormalities, immune dysregulation has also been implicated in the progression of MAFLD, whereby alterations in T cell subset proportions and upregulation of proinflammatory cytokines may exacerbate the hepatic inflammatory microenvironment, forming a vicious cycle with lipid deposition (44). Moreover, accumulating evidence suggests that gut microbiota dysbiosis and impaired intestinal barrier function may influence lipid metabolism and inflammatory status through the gut–liver axis; however, the specific mechanistic pathways underlying their role in the association between AIP and MAFLD remain to be elucidated (45, 46). Overall, These pathological processes closely align with the concept of metabolically driven liver disease emphasized by MAFLD and provide biological plausibility for the robust associations observed in this study.

Compared with the meta-analysis published by Ismaiel et al. in 2022 that focused on NAFLD and AIP (33), the present study extends and refines the existing evidence in several important aspects. First, both MAFLD and NAFLD studies were included, and subgroup analyses were conducted according to diagnostic frameworks. Studies using MAFLD definitions showed lower internal heterogeneity and more stable effect estimates, suggesting that under diagnostic systems emphasizing metabolic dysfunction, the effect of AIP as an integrated metabolic–lipid marker is more readily observed. Second, this study not only pooled association measures such as odds ratios and hazard ratios but also systematically evaluated differences in AIP levels (WMD) and diagnostic performance (AUC), thereby constructing a more comprehensive evidence framework spanning risk association, phenotypic differences, and diagnostic utility. Third, the sample size and study design diversity exceeded previous analyses, encompassing general populations and those with obesity, type 2 diabetes mellitus, and pregnancy, thereby enhancing generalizability. Finally, through structured subgroup and meta-regression analyses, this study sought to address which populations and metabolic contexts may derive greater utility from AIP, rather than limiting the analysis to whether an association exists.

Several noteworthy differences and unexpected findings were also observed. The association between AIP and NAFLD or MAFLD was stronger and accompanied by lower heterogeneity in populations with BMI of at least 25 kg/m^2^, whereas in populations with BMI below 25 kg/m^2^ the effect size was slightly attenuated but remained statistically significant. This finding indicates that AIP is not restricted to populations with obesity but reflects metabolic lipid abnormalities across different body weight spectra, with amplification of the association under conditions of obesity. AIP was significantly associated with NAFLD/MAFLD in non-diabetic populations, whereas the type 2 diabetes mellitus subgroup showed a confidence interval crossing unity without statistical significance. This result may be related to the limited number of available studies and sample size in this subgroup, as well as to wider confidence intervals produced by random-effects models in the presence of heterogeneity. It also suggests that in settings of substantial metabolic burden, the marginal predictive value of AIP may be partially obscured by other strong risk factors. In addition, in some younger populations the effect size and diagnostic performance of AIP were slightly higher than in older populations, indicating that this index may reflect adverse metabolic phenotypes at relatively early stages before overt clinical events occur, which may have implications for earlier screening strategies.

From a conceptual perspective, the present findings not only support the view that MAFLD represents a systemic metabolic disease but also provide evidence for the development of an integrated liver–cardiovascular–metabolic risk assessment framework. The stable association between AIP and NAFLD or MAFLD, together with its moderate diagnostic performance, indicates that a simple index derived from routine lipid measurements can simultaneously signal hepatic fat accumulation and atherosclerotic risk. In this context, fatty liver disease can be viewed not merely as a localized hepatic phenotype but as a sentinel marker within cardiometabolic risk stratification. Large cohort data linking AIP to all-cause and cardiovascular mortality in MAFLD patients support incorporating this index into risk stratification frameworks for identifying those at elevated hepatic and cardiovascular risk (47, 48), AIP has potential to be incorporated into comprehensive risk scoring systems for MAFLD to identify subgroups with concomitantly elevated hepatic and cardiovascular risk. Compared with individual lipid parameters such as triglycerides or HDL-C alone, AIP captures the degree of overall lipid profile imbalance and may more closely reflect the shared metabolic, inflammatory, and atherosclerotic processes underlying MAFLD (7, 9, 49). By integrating evidence across multiple outcomes and populations, this study advances the conceptualization of AIP from a single lipid-derived marker to a cross-organ metabolic signal. At the clinical level, AIP, as a simple index calculated from routine lipid parameters, offers the advantages of easy accessibility, low cost, and good reproducibility, and may serve as an auxiliary tool for early risk screening in MAFLD populations and cardiovascular comorbidity stratification. If combined with standardized thresholds and prospective follow-up data in the future, its incremental value in comprehensive risk assessment models could be further clarified.

The I^2^ values for the primary outcomes in the present study all exceeded 95%, indicating substantial and complex heterogeneity across studies. Given that AIP itself is closely associated with multiple metabolic abnormalities, differences among studies in diagnostic frameworks, outcome ascertainment methods, and the metabolic profiles of study populations may have influenced the effect estimates. Furthermore, although we preferentially extracted the most fully adjusted multivariable model from each study, the extent of confounder control varied across studies — some adjusted only for age and sex, while others additionally included metabolic indicators such as BMI, blood glucose, blood pressure, or insulin resistance — and such disparities in adjustment strategies may have amplified between-study variability at the statistical level. Although subgroup analyses and meta-regression were performed to explore potential sources of heterogeneity, and heterogeneity was reduced in certain strata, residual differences that could not be fully explained persisted, suggesting the existence of unmeasured or inadequately reported effect modifiers. Importantly, the direction of effects across studies was broadly consistent, with no directional conflicts observed, and sensitivity analyses demonstrated that sequential exclusion of individual studies did not materially alter the pooled results. Accordingly, a random-effects model was adopted to yield more conservative estimates, and in interpreting the results, we emphasize that the observed heterogeneity more likely reflects variation in the magnitude of the effect across different study settings rather than instability of the association itself.

Several limitations warrant consideration. First, most included studies were cross-sectional in design, which precludes determination of causal direction. Whether elevated AIP serves as a MAFLD precursor or reflects long-standing metabolic dysfunction remains unclear, requiring clarification through large-scale prospective cohort and interventional studies. Second, most studies did not use liver histology as the reference standard, which may lead to underestimation of mild steatosis and affect precision of effect estimates. Residual confounding from unmeasured or inadequately adjusted variables, including dietary intake, physical activity, and medication use, may have affected the findings. Third, grey literature and unpublished studies were not included in the present study. Although Egger’s test did not indicate significant publication bias, the possibility of selective reporting cannot be entirely excluded. Fourth, due to the lack of individual-level data, we were unable to conduct more granular stratified analyses, such as those by different AIP quartiles, sex-specific effects, or metabolic profile subgroups, thereby limiting the precise extrapolation of the results to specific populations. While these limitations restrict causal conclusions and generalizability to specific populations, they do not detract from the principal observation that elevated AIP is significantly associated with heightened NAFLD/MAFLD risk. Instead, they highlight priorities for future research, including validation across different metabolic backgrounds and age groups, incorporation of histological outcomes, multicenter prospective follow-up, and evaluation of multivariable integrated models to determine the incremental contribution of AIP to clinical decision-making.

## 5 Conclusions

The present systematic review identifies significant correlations between elevated AIP and heightened MAFLD/NAFLD risk, alongside moderate diagnostic accuracy. These findings support consideration of AIP as an important link between lipid metabolic abnormalities and MAFLD. Future studies should clarify the causal relationships between AIP and the occurrence, progression, and cardiovascular outcomes of MAFLD in larger, multiethnic, and multicenter cohorts, refine population-specific cutoff values, and evaluate the incremental clinical value of incorporating AIP into comprehensive risk prediction models with respect to clinical outcomes, follow-up strategies, and intervention decisions, thereby advancing more precise and stratified management of MAFLD and related cardiometabolic comorbidities.

## Data Availability

The original contributions presented in the study are included in the article/**Supplementary Material**. Further inquiries can be directed to the corresponding authors.
