# Supplementary material for: Association between the atherogenic index of plasma and metabolic-associated/nonalcoholic fatty liver disease: a systematic review and meta-analysis
Source: Front Endocrinol (Lausanne). 2026 Mar 31;17:1800577. doi: 10.3389/fendo.2026.1800577 (PMC13076180; doi:10.3389/fendo.2026.1800577)
Supplement: Supplementary file 1 [file DataSheet1.docx]

**Table S1** Search strategy for Pubmed, Embase, Web of Science, Cochrane

| **Database** | **Search-detail** | **results** | **Time** |
| --- | --- | --- | --- |
| Pubmed | (("Fatty Liver"[Mesh]) OR (Fatty Liver[Title/Abstract] OR fat liver[Title/Abstract] OR fatty change in liver[Title/Abstract] OR fatty hepatic infiltration[Title/Abstract] OR fatty infiltration of liver[Title/Abstract] OR fatty infiltration of the liver[Title/Abstract] OR fatty liver disease[Title/Abstract] OR fatty liver infiltration[Title/Abstract] OR fatty liver syndrome[Title/Abstract] OR fatty metamorphosis of the liver[Title/Abstract] OR hepatic fatty infiltration[Title/Abstract] OR hepatic lipidosis[Title/Abstract] OR hepatic lipomatosis[Title/Abstract] OR hepatic lipomatous lesion[Title/Abstract] OR hepatic steatosis[Title/Abstract] OR hepato steatosis[Title/Abstract] OR hepatolipidosis[Title/Abstract] OR hepatosteatosis[Title/Abstract] OR Liver Fatty[Title/Abstract] OR liver fatty change[Title/Abstract] OR liver fatty infiltration[Title/Abstract] OR liver fatty replacement[Title/Abstract] OR liver lipidosis[Title/Abstract] OR liver lipomatosis[Title/Abstract] OR Liver Steatoses[Title/Abstract] OR Liver Steatosis[Title/Abstract] OR MAFLD[Title/Abstract] OR Metabolic Dysfunction Associated Fatty Liver Disease[Title/Abstract] OR NAFLD[Title/Abstract] OR Non alcoholic Fatty Liver Disease[Title/Abstract] OR steato hepatosis[Title/Abstract] OR Steatohepatitides[Title/Abstract] OR Steatohepatitis[Title/Abstract] OR steatohepatopathy[Title/Abstract] OR steatohepatosis[Title/Abstract] OR Steatoses Liver[Title/Abstract] OR Steatoses Visceral[Title/Abstract] OR Steatosis Liver[Title/Abstract] OR Steatosis of Liver[Title/Abstract] OR Steatosis Visceral[Title/Abstract] OR steatotic hepatopathy[Title/Abstract] OR steatotic liver[Title/Abstract] OR Visceral Steatoses[Title/Abstract] OR Visceral Steatosis[Title/Abstract])) AND (atherogenic index[Title/Abstract] OR atherogen index of plasma[Title/Abstract] OR atherogenic index of plasma[Title/Abstract] OR atherogenicity index[Title/Abstract] OR atherogenicity index of plasma[Title/Abstract] OR atherogenic index[Title/Abstract]) | 116 | 2025.10.12 |
| Embase | ('atherogenic index'/exp OR 'atherogen index of plasma':ab,ti OR 'atherogenic index of plasma':ab,ti OR 'atherogenicity index':ab,ti OR 'atherogenicity index of plasma':ab,ti OR 'atherogenic index':ab,ti) AND ('fatty liver'/exp OR 'fatty liver':ab,ti OR 'fat liver':ab,ti OR 'fatty change in liver':ab,ti OR 'fatty hepatic infiltration':ab,ti OR 'fatty infiltration of liver':ab,ti OR 'fatty infiltration of the liver':ab,ti OR 'fatty liver disease':ab,ti OR 'fatty liver infiltration':ab,ti OR 'fatty liver syndrome':ab,ti OR 'fatty metamorphosis of the liver':ab,ti OR 'hepatic fatty infiltration':ab,ti OR 'hepatic lipidosis':ab,ti OR 'hepatic lipomatosis':ab,ti OR 'hepatic lipomatous lesion':ab,ti OR 'hepatic steatosis':ab,ti OR 'hepato steatosis':ab,ti OR hepatolipidosis:ab,ti OR hepatosteatosis:ab,ti OR 'liver fatty':ab,ti OR 'liver fatty change':ab,ti OR 'liver fatty infiltration':ab,ti OR 'liver fatty replacement':ab,ti OR 'liver lipidosis':ab,ti OR 'liver lipomatosis':ab,ti OR 'liver steatoses':ab,ti OR 'liver steatosis':ab,ti OR mafld:ab,ti OR 'metabolic dysfunction associated fatty liver disease':ab,ti OR nafld:ab,ti OR 'non alcoholic fatty liver disease':ab,ti OR 'steato hepatosis':ab,ti OR steatohepatitides:ab,ti OR steatohepatitis:ab,ti OR steatohepatopathy:ab,ti OR steatohepatosis:ab,ti OR 'steatoses liver':ab,ti OR 'steatoses visceral':ab,ti OR 'steatosis liver':ab,ti OR 'steatosis of liver':ab,ti OR 'steatosis visceral':ab,ti OR 'steatotic hepatopathy':ab,ti OR 'steatotic liver':ab,ti OR 'visceral steatoses':ab,ti OR 'visceral steatosis':ab,ti) | 210 | 2025.10.12 |
| Web of Science | Fatty Liver OR fat liver OR fatty change in liver OR fatty hepatic infiltration OR fatty infiltration of liver OR fatty infiltration of the liver OR fatty liver disease OR fatty liver infiltration OR fatty liver syndrome OR fatty metamorphosis of the liver OR hepatic fatty infiltration OR hepatic lipidosis OR hepatic lipomatosis OR hepatic lipomatous lesion OR hepatic steatosis OR hepato steatosis OR hepatolipidosis OR hepatosteatosis OR Liver Fatty OR liver fatty change OR liver fatty infiltration OR liver fatty replacement OR liver lipidosis OR liver lipomatosis OR Liver Steatoses OR Liver Steatosis OR MAFLD OR Metabolic Dysfunction Associated Fatty Liver Disease OR NAFLD OR Non alcoholic Fatty Liver Disease OR steato hepatosis OR Steatohepatitides OR Steatohepatitis OR steatohepatopathy OR steatohepatosis OR Steatoses Liver OR Steatoses Visceral OR Steatosis Liver OR Steatosis of Liver OR Steatosis Visceral OR steatotic hepatopathy OR steatotic liver OR Visceral Steatoses OR Visceral Steatosis (Topic) and atherogenic index OR atherogen index of plasma OR atherogenic index of plasma OR atherogenicity index OR atherogenicity index of plasma OR atherogenic index (Topic) | 287 | 2025.10.12 |
| Cochrane | (atherogenic index OR atherogen index of plasma OR atherogenic index of plasma OR atherogenicity index OR atherogenicity index of plasma OR atherogenic index):ti,ab,kw AND (MeSH descriptor: [Fatty Liver] explode all trees OR (Fatty Liver OR fat liver OR fatty change in liver OR fatty hepatic infiltration OR fatty infiltration of liver OR fatty infiltration of the liver OR fatty liver disease OR fatty liver infiltration OR fatty liver syndrome OR fatty metamorphosis of the liver OR hepatic fatty infiltration OR hepatic lipidosis OR hepatic lipomatosis OR hepatic lipomatous lesion OR hepatic steatosis OR hepato steatosis OR hepatolipidosis OR hepatosteatosis OR Liver Fatty OR liver fatty change OR liver fatty infiltration OR liver fatty replacement OR liver lipidosis OR liver lipomatosis OR Liver Steatoses OR Liver Steatosis OR MAFLD OR Metabolic Dysfunction Associated Fatty Liver Disease OR NAFLD OR Non alcoholic Fatty Liver Disease OR steato hepatosis OR Steatohepatitides OR Steatohepatitis OR steatohepatopathy OR steatohepatosis OR Steatoses Liver OR Steatoses Visceral OR Steatosis Liver OR Steatosis of Liver OR Steatosis Visceral OR steatotic hepatopathy OR steatotic liver OR Visceral Steatoses OR Visceral Steatosis):ti,ab,kw) | 676 | 2025.10.12 |

**Table S2** NIH--Literature quality assessment (Please refer to the Excel sheet)

| study |  | 1.Was the research question or objective in this paper clearly stated? | 2.Was the study population clearly specified and defined? | 3.Was the participation rate of eligible persons at least 50%? | 4.Were all the subjects selected or recruited from the same or similar populations (including the same time period)? Were inclusion and exclusion criteria for being in the study prespecified and applied uniformly to all participants? | 5.Was a sample size justification, power description, or variance and effect estimates provided? | 6.For the analyses in this paper, were the exposure(s) of interest measured prior to the outcome(s) being measured?【Cross-sectional study does not require answering】 | 7.Was the timeframe sufficient so that one could reasonably expect to see an association between exposure and outcome if it existed?【Cross-sectional study does not require answering】 | 8.For exposures that can vary in amount or level, did the study examine different levels of the exposure as related to the outcome (e.g., categories of exposure, or exposure measured as continuous variable)? | 9.Were the exposure measures (independent variables) clearly defined, valid, reliable, and implemented consistently across all study participants? | 10.Was the exposure(s) assessed more than once over time?【Cross-sectional study does not require answering】 | 11.Were the outcome measures (dependent variables) clearly defined, valid, reliable, and implemented consistently across all study participants? | 12.Were the outcome assessors blinded to the exposure status of participants? | 13.Was loss to follow-up after baseline 20% or less?【Cross-sectional study does not require answering】 | 14.Were key potential confounding variables measured and adjusted statistically for their impact on the relationship between exposure(s) and outcome(s)? |  |  |
| --- | --- | --- | --- | --- | --- | --- | --- | --- | --- | --- | --- | --- | --- | --- | --- | --- | --- |
| 28 | Xie 2019 | √ | √ | ❌ | √ | ❓ (NR) | NA | NA | √ | √ | NA | √ | √ | NA | √ | fair | cross-sectional study |
| 34 | Nida 2025 | √ | √ | ❌ | √ | √ | NA | NA | √ | √ | NA | √ | √ | NA | √ | good | cross-sectional study |
| 23 | Song 2025 | √ | √ | ❌ | √ | ❓ (NR) | √ | √ | √ | √ | ❌ | √ | √ | ✔️√ | √ | good | cohort study |
| 26 | Duan 2022 | √ | √ | ❌ | √ | ❓ (NR) | NA | NA | √ | √ | NA | √ | √ | NA | √ | good | cross-sectional study |
| 27 | Matteis 2025 | √ | √ | ❌ | √ | ❓ (NR) | NA | NA | √ | √ | NA | √ | √ | NA | √ | fair | cross-sectional study |
| 32 | Ciftel 2024 | √ | √ | ❌ | √ | ❓ (NR) | NA | NA | √ | √ | NA | √ | √ | NA | √ | fair | cross-sectional study |
| 40 | chen 2024 | √ | √ | ❌ | √ | ❓ (NR) | NA | NA | √ | √ | NA | √ | √ | NA | √ | good | cross-sectional study |
| 60 | Efrem 2022 | √ | √ | ❌ | √ | ❓ (NR) | NA | NA | √ | √ | NA | √ | √ | NA | √ | good | cross-sectional study |
| 35 | Xie 2021 | √ | √ | ❌ | √ | ❓ (NR) | NA | NA | √ | √ | NA | √ | √ | NA | √ | fair | cross-sectional study |
| 36 | Lin 2022 | √ | √ | ❌ | √ | ❓ (NR) | NA | NA | √ | √ | NA | √ | √ | NA | √ | fair | cross-sectional study |
| 37 | Peng 2023 | √ | √ | ❌ | √ | ❓ (NR) | NA | NA | √ | √ | NA | √ | √ | NA | √ | good | cross-sectional study |
| 44 | Lee 2006 | √ | √ | ❌ | √ | ❓ (NR) | NA | NA | √ | √ | NA | √ | √ | NA | ❌ | fair | cross-sectional study |
| 47 | Ruan 2024 | √ | √ | ❌ | √ | ❓ (NR) | NA | NA | √ | √ | NA | √ | √ | NA | √ | fair | cross-sectional study |
| 53 | Mohammedsaeed 2025 | √ | √ | ❌ | √ | ❓ (NR) | NA | NA | √ | √ | NA | √ | √ | NA | √ | good | cross-sectional study |
| 57 | Turecký 2022 | √ | √ | ❌ | √ | ❓ (NR) | NA | NA | √ | √ | NA | √ | √ | NA | ❌ | fair | cross-sectional study |
| 64 | Dong 2020 | √ | √ | ❌ | √ | ❓ (NR) | NA | NA | √ | √ | NA | √ | √ | NA | √ | good | cross-sectional study |
| 16 | Shuai 2025 | √ | √ | ❌ | √ | ❓ (NR) | NA | NA | √ | √ | NA | √ | √ | NA | √ | fair | cross-sectional study |
| 19 | Fadaei 2018 | √ | √ | ❌ | √ | ❓ (NR) | NA | NA | √ | √ | NA | √ | √ | NA | √ | good | cross-sectional study |
| 24 | Wang 2025 | √ | √ | ❌ | √ | ❓ (NR) | NA | NA | √ | √ | NA | √ | √ | NA | √ | fair | cross-sectional study |
| 29 | wang 2018 | √ | √ | ❌ | √ | ❓ (NR) | NA | NA | √ | √ | NA | √ | √ | NA | √ | fair | cross-sectional study |
| 30 | Samimi 2022 | √ | √ | ❓ (NR) | ❌ | √ | √ | √ | √ | √ | ❌ | √ | ❌ | - | - | fair | case control study |
| 31 | Liu 2022 | √ | √ | ❌ | √ | ❓ (NR) | NA | NA | √ | √ | NA | √ | √ | NA | √ | fair | cross-sectional study |


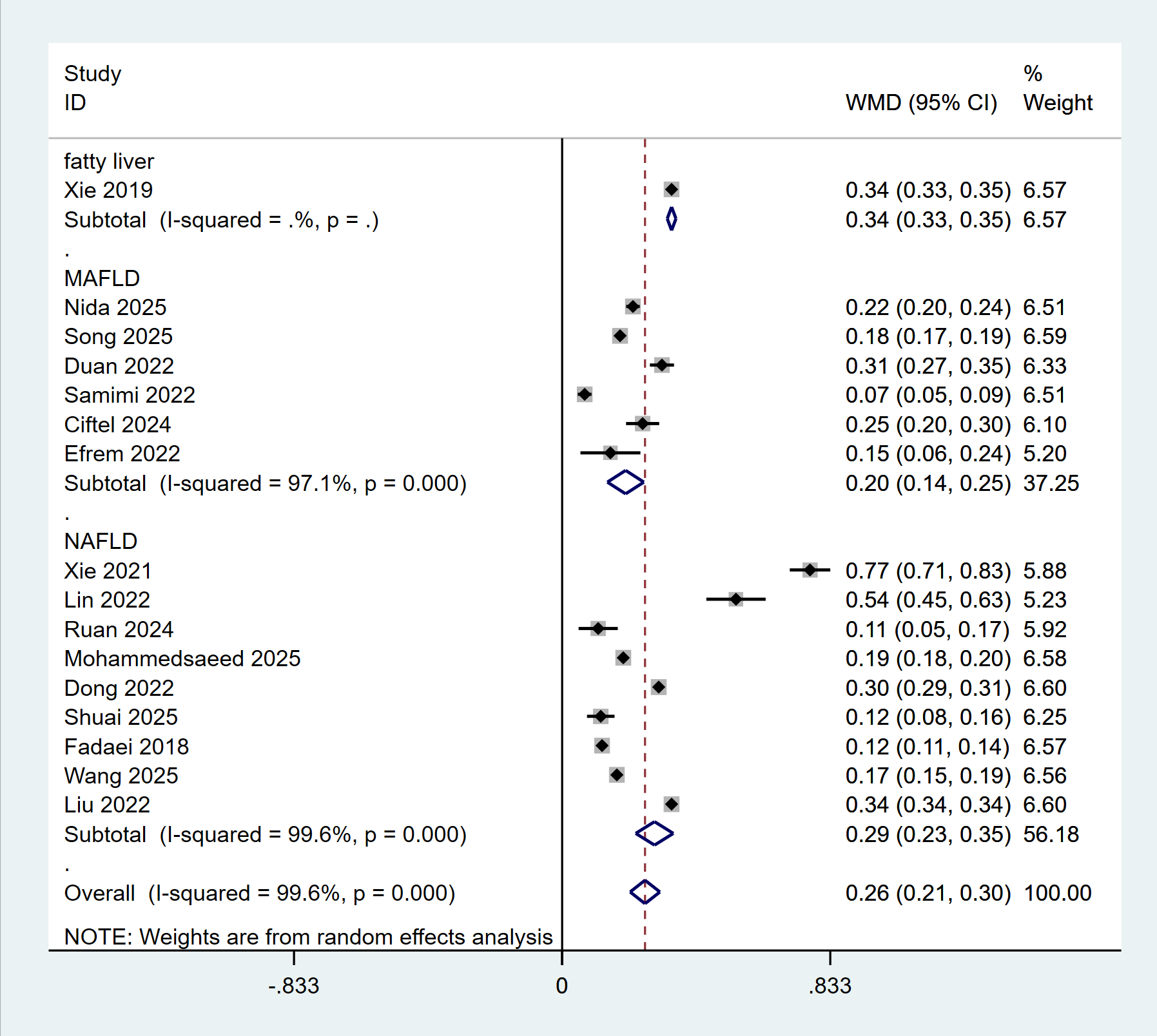


**Figure S1** The Difference in AIP levels by diagnostic criteria subgroup


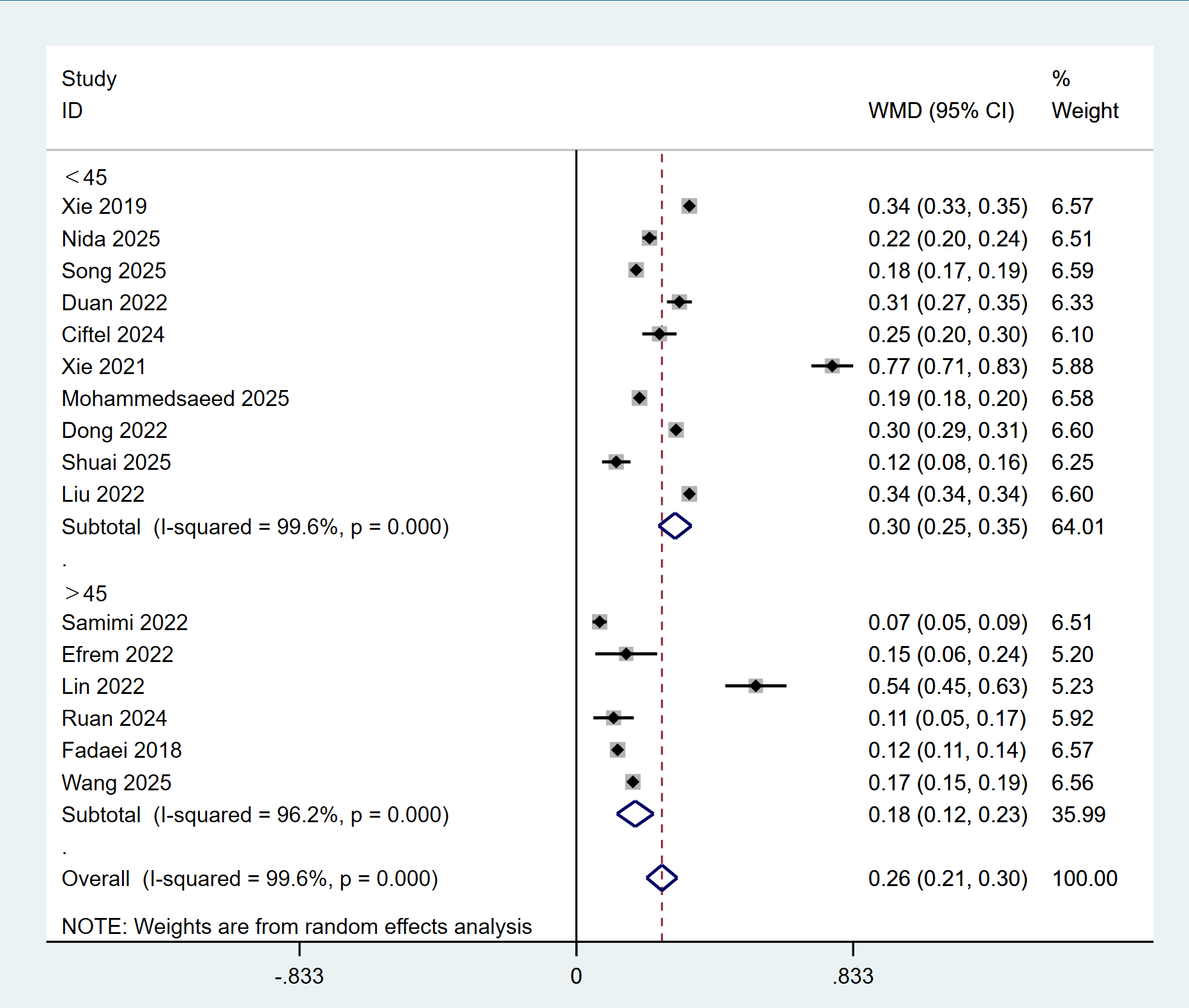


**Figure S2** The Difference in AIP levels by age subgroup


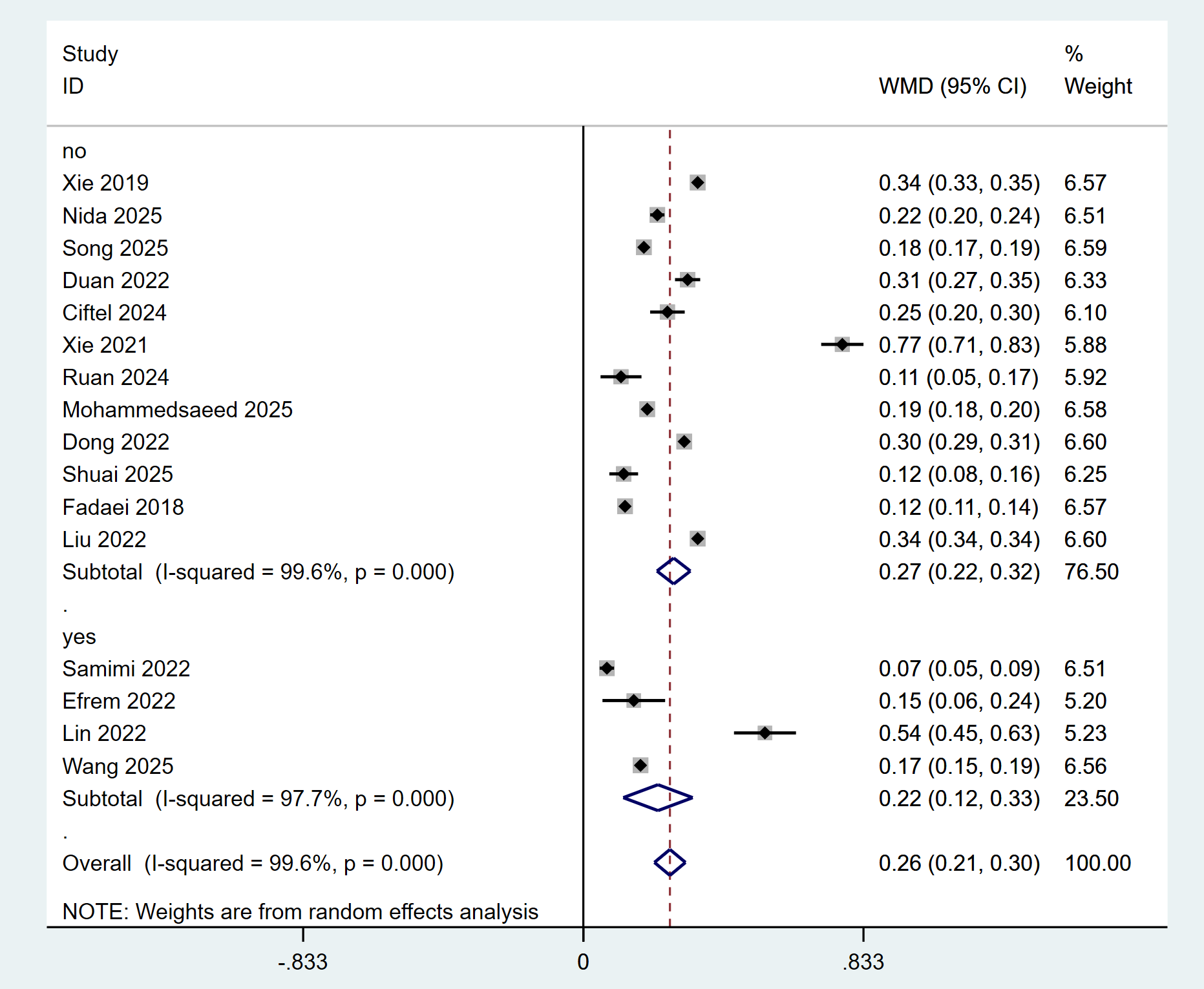


**Figure S3** The Difference in AIP levels by type 2 diabetes subgroup


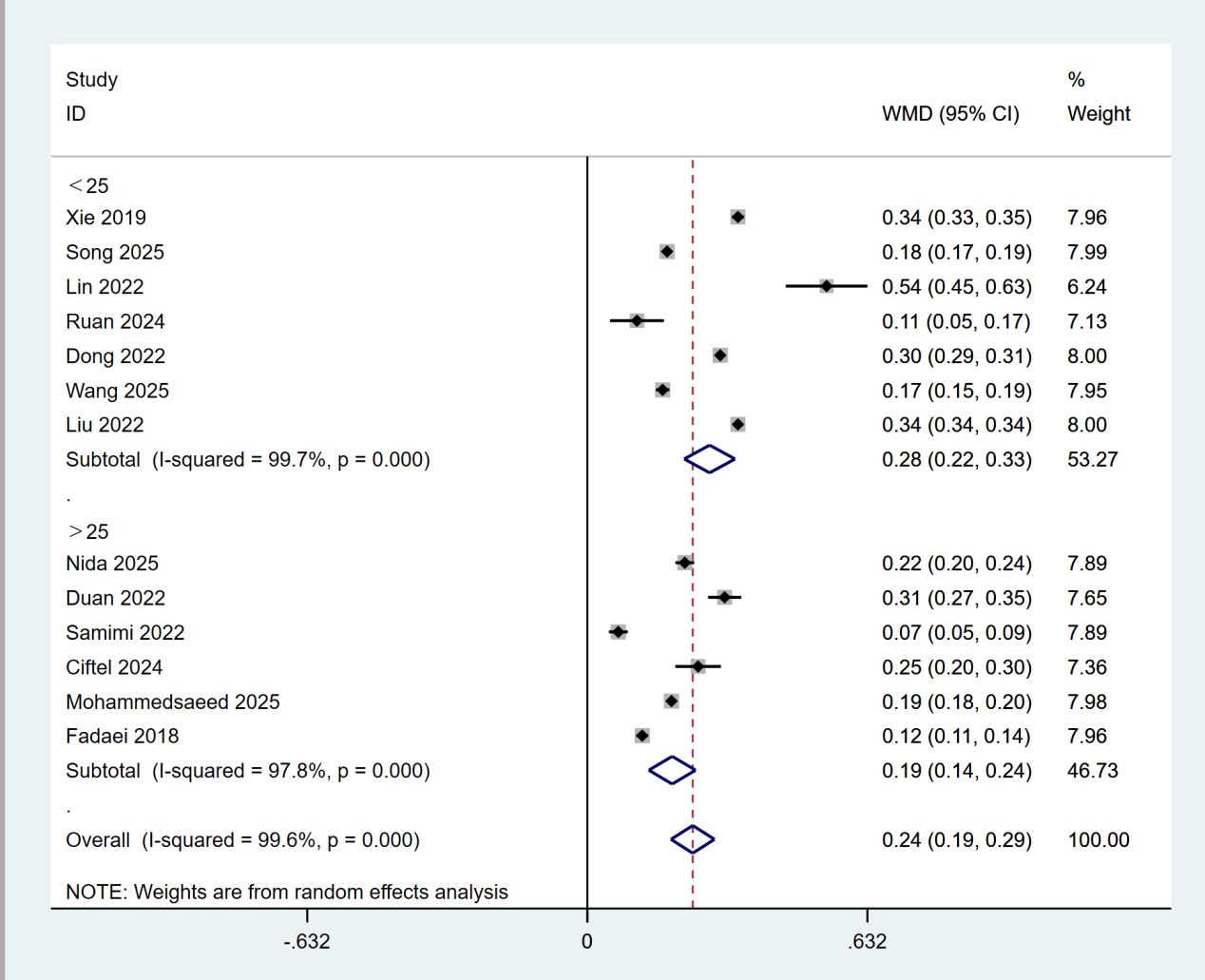


**Figure S4** The Difference in AIP levels by BMI subgroup


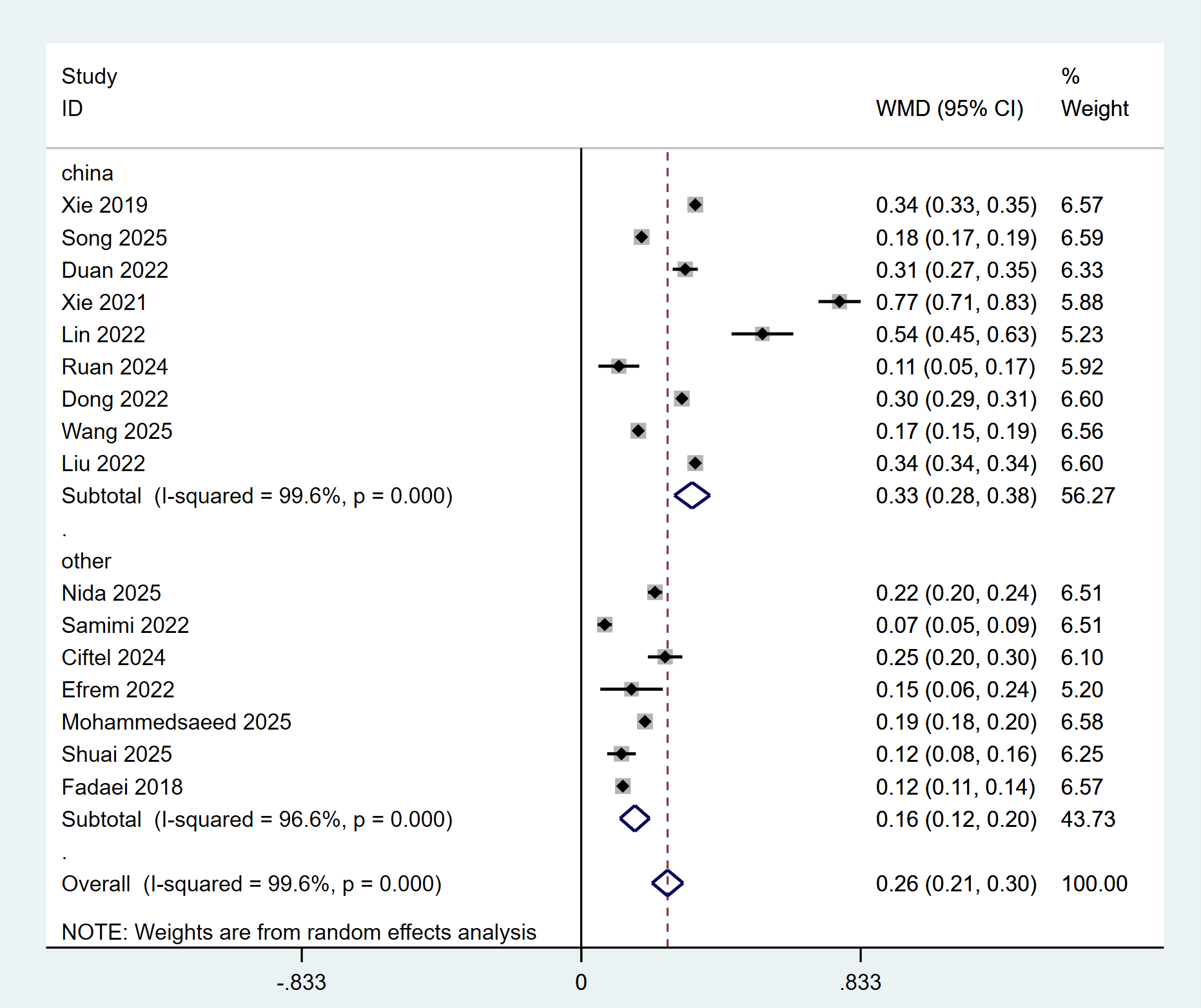


**Figure S5** The Difference in AIP levels by area subgroup


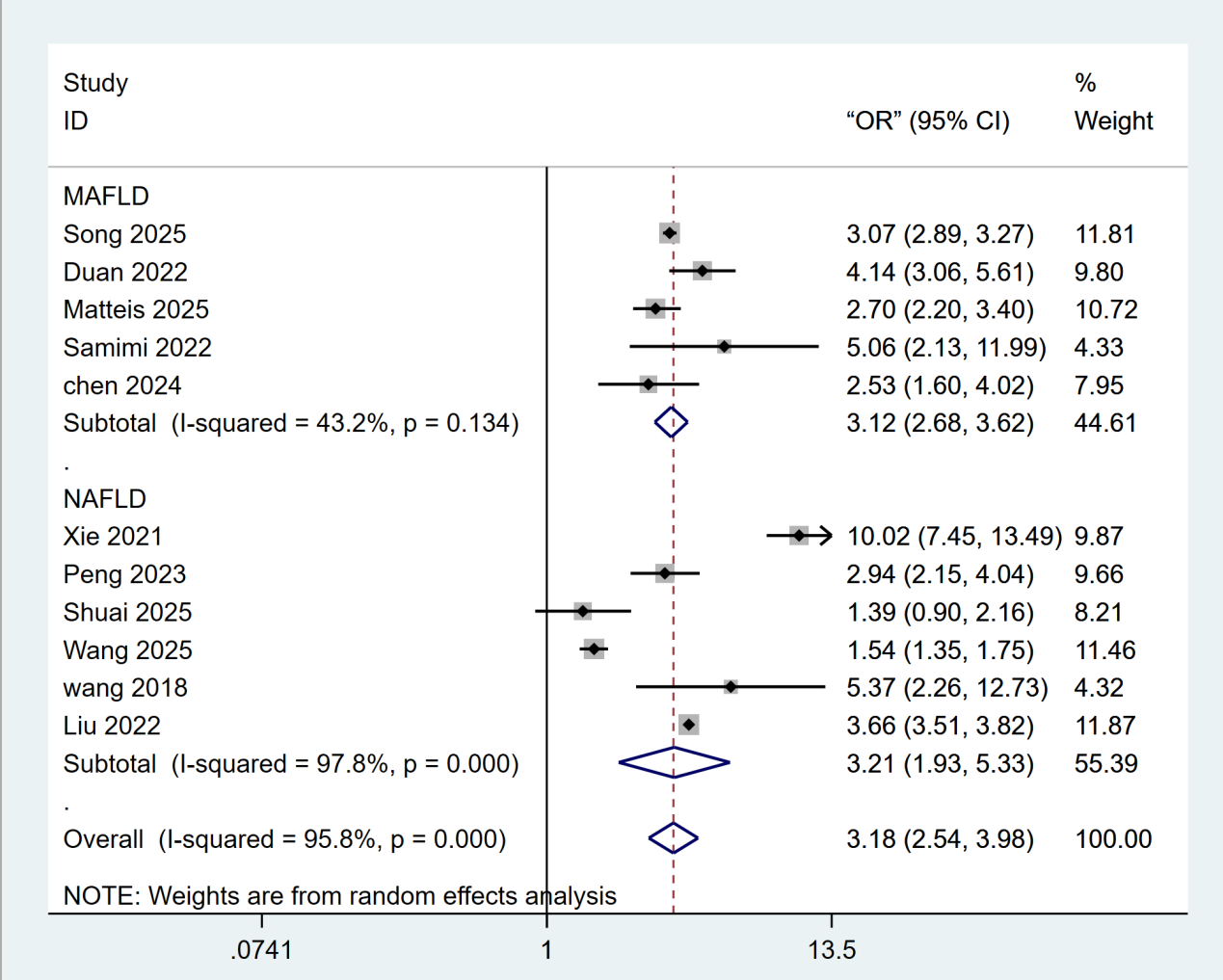


**Figure S6** The association between AIP and NAFLD/MAFLD by diagnostic criteria subgroup


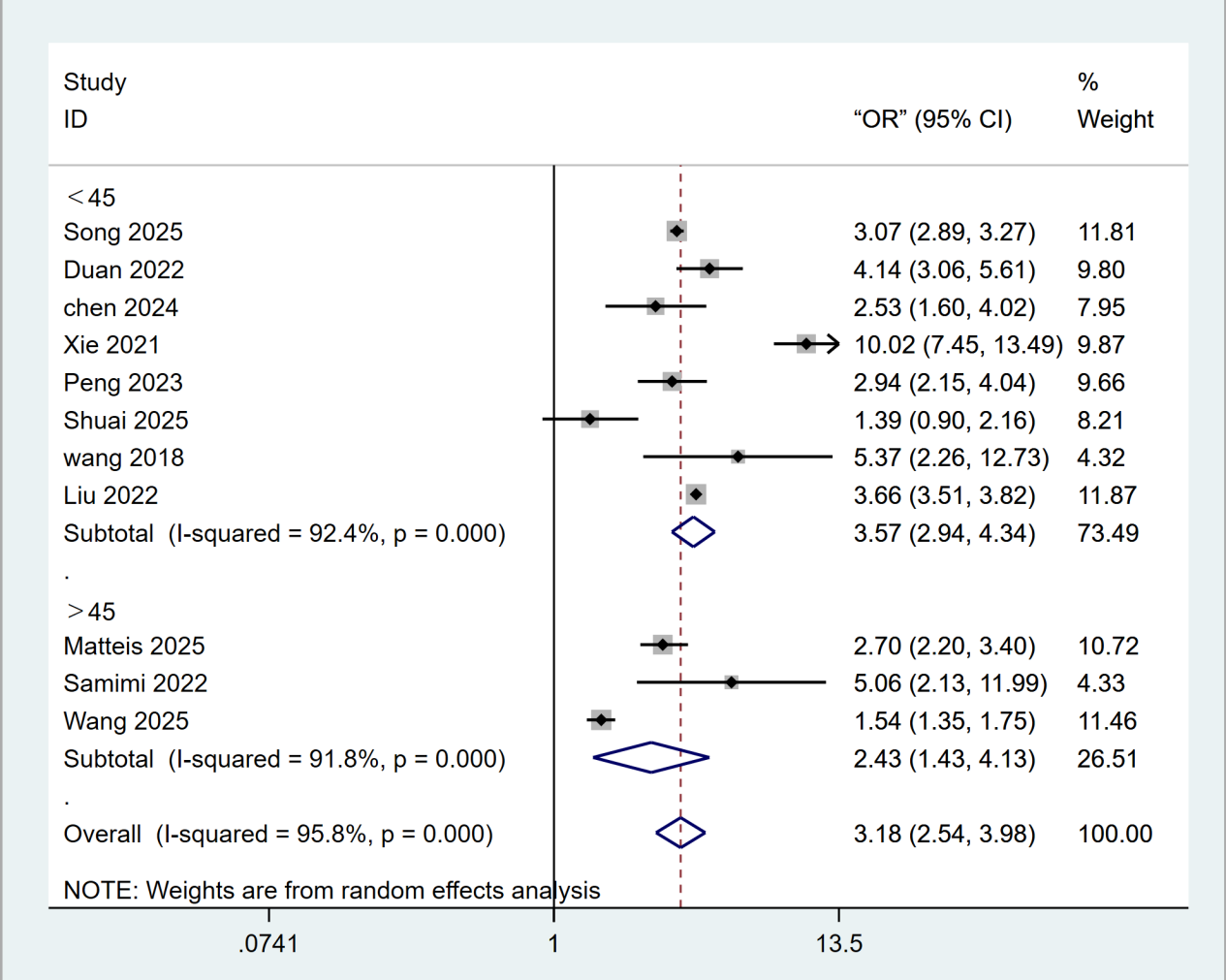


**Figure S7** The association between AIP and NAFLD/MAFLD by age subgroup


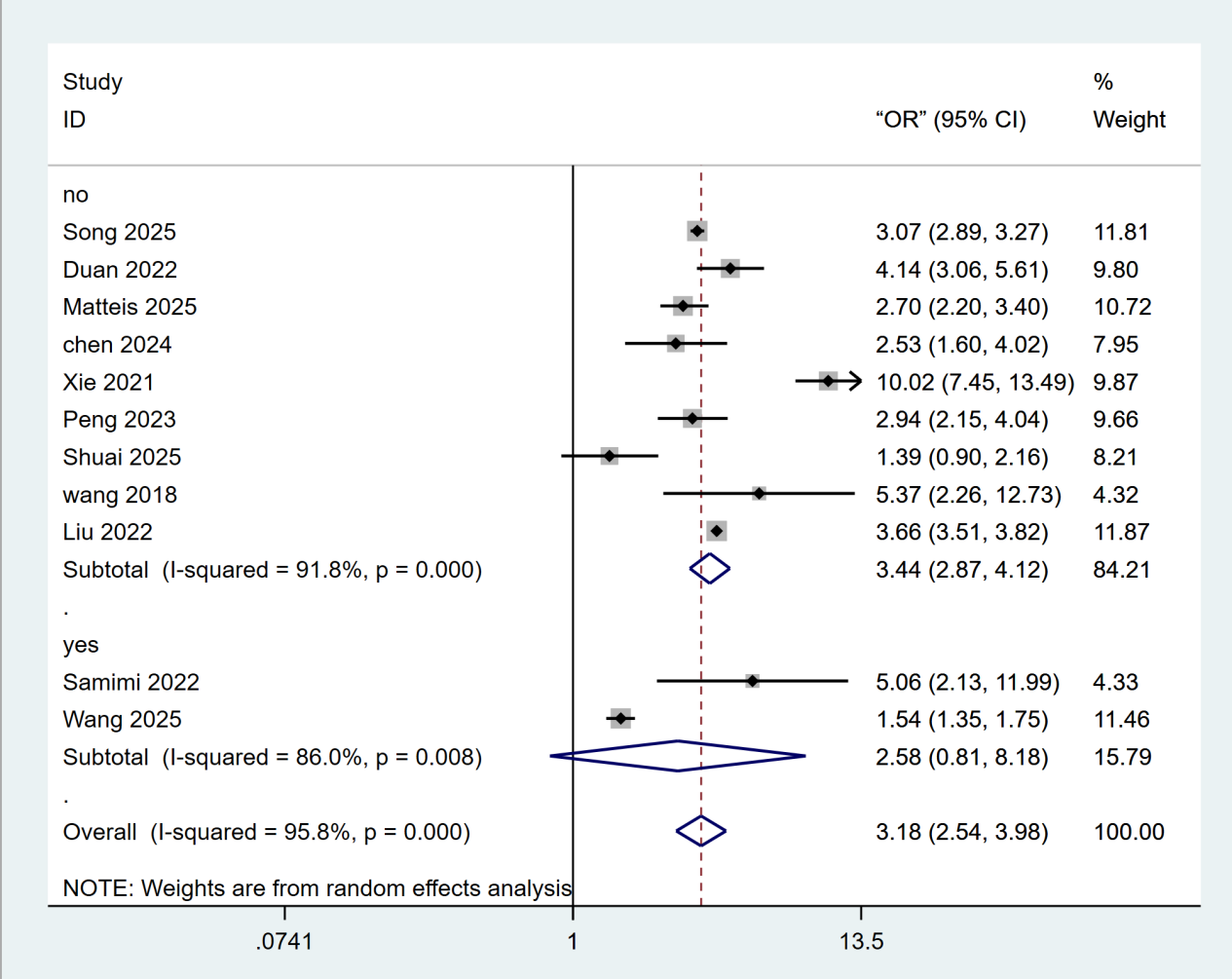
**Figure S8** The association between AIP and NAFLD/MAFLD by type 2 diabetes subgroup


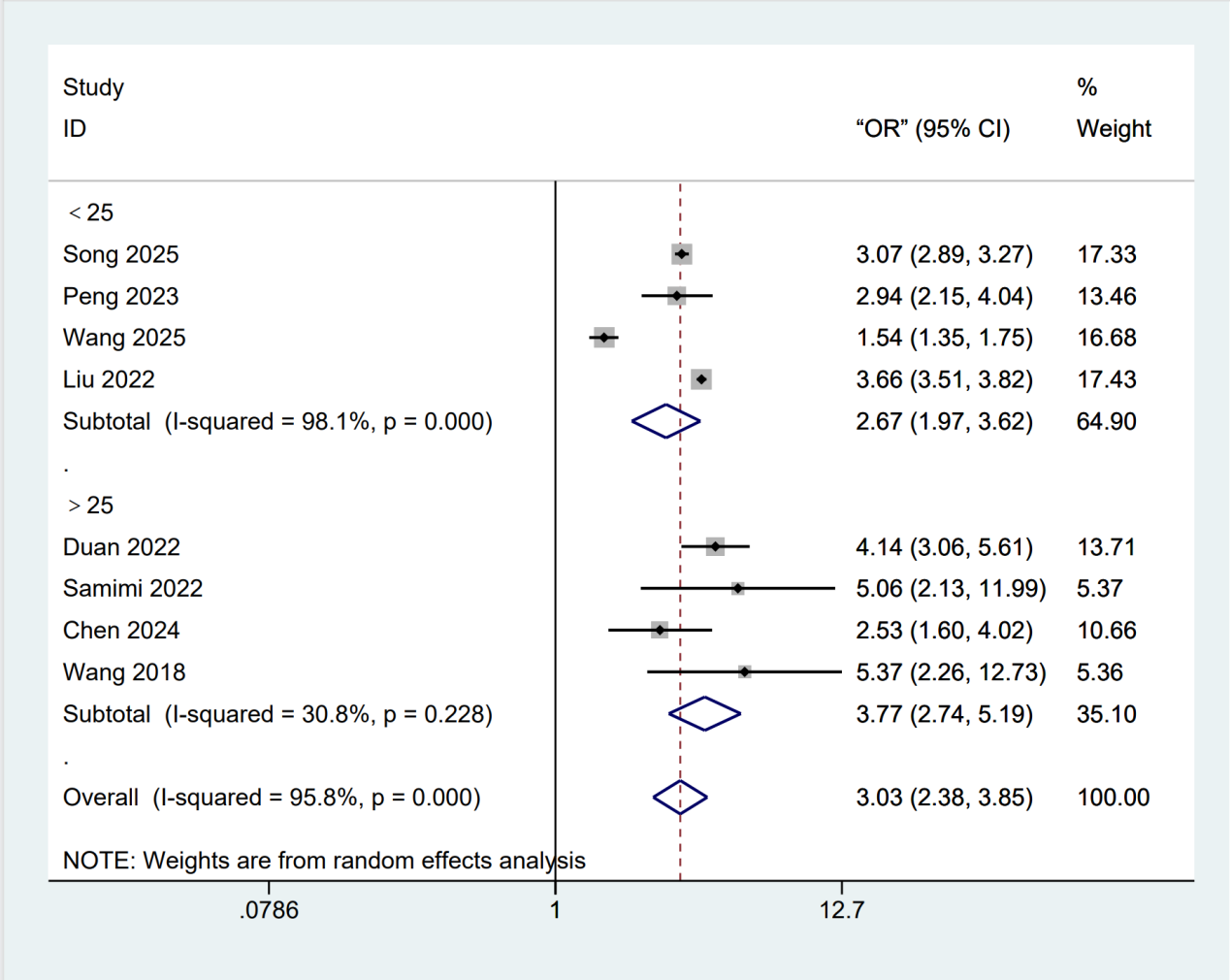
**Figure S9** The association between AIP and NAFLD/MAFLD by BMI subgroup


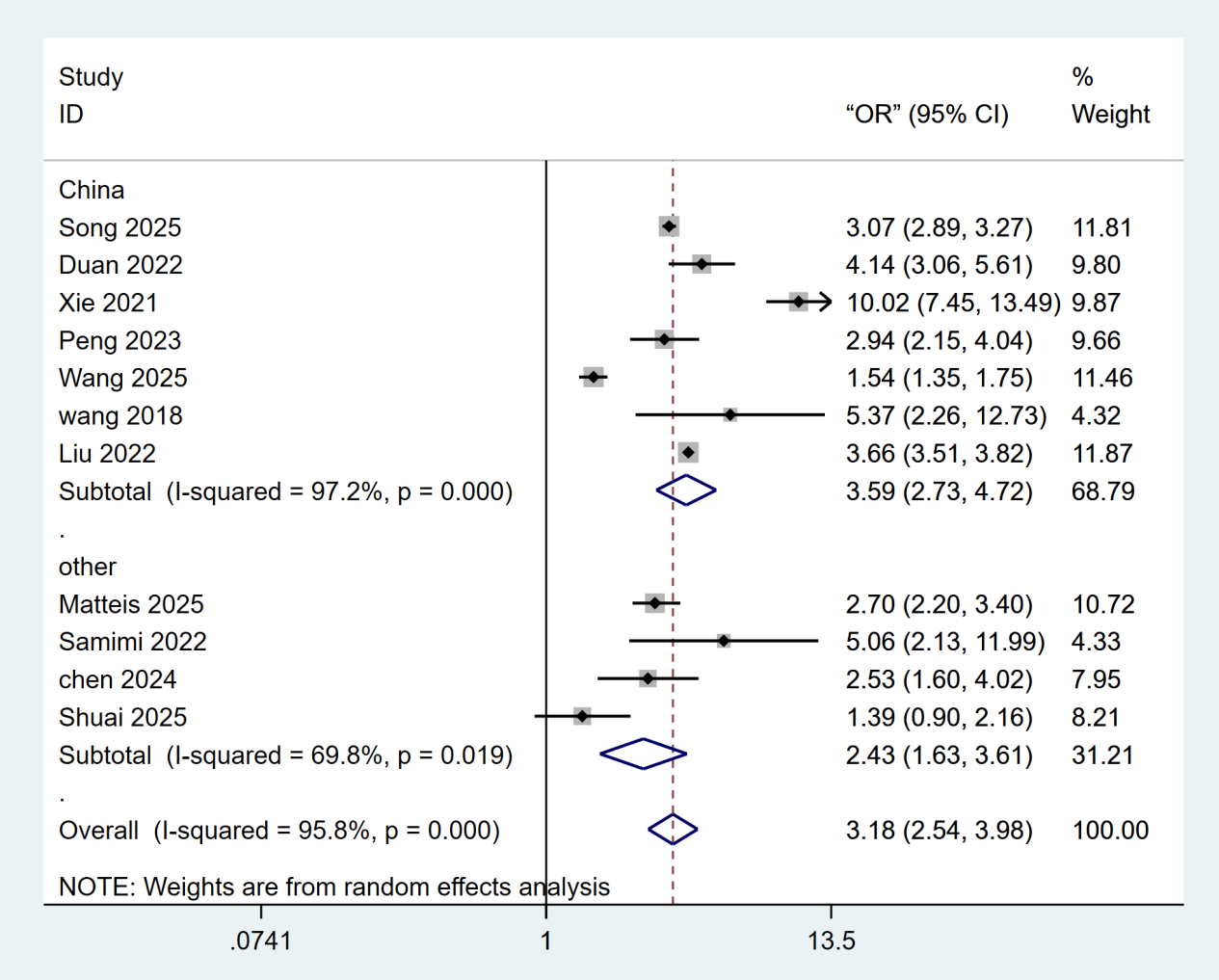
**Figure S10** The association between AIP and NAFLD/MAFLD by area subgroup

1. By age


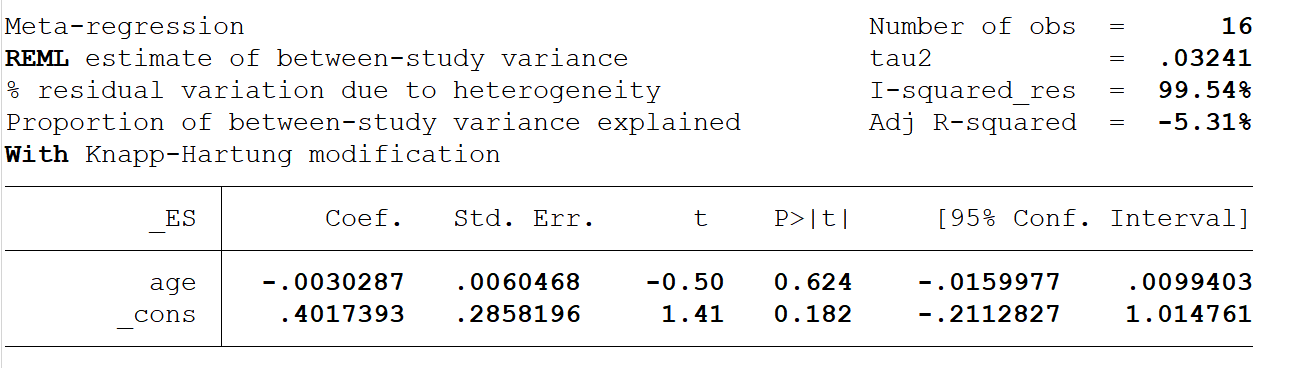


(2) By Sex ratio


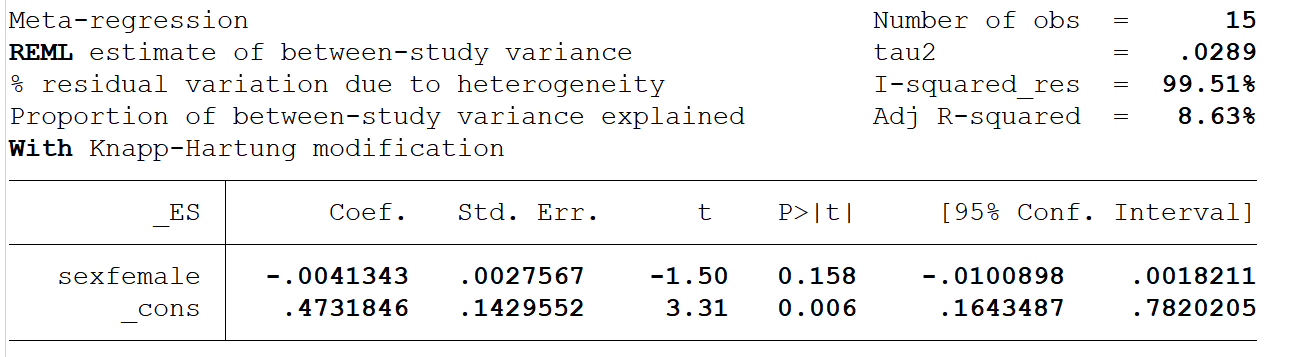


1. By BMI


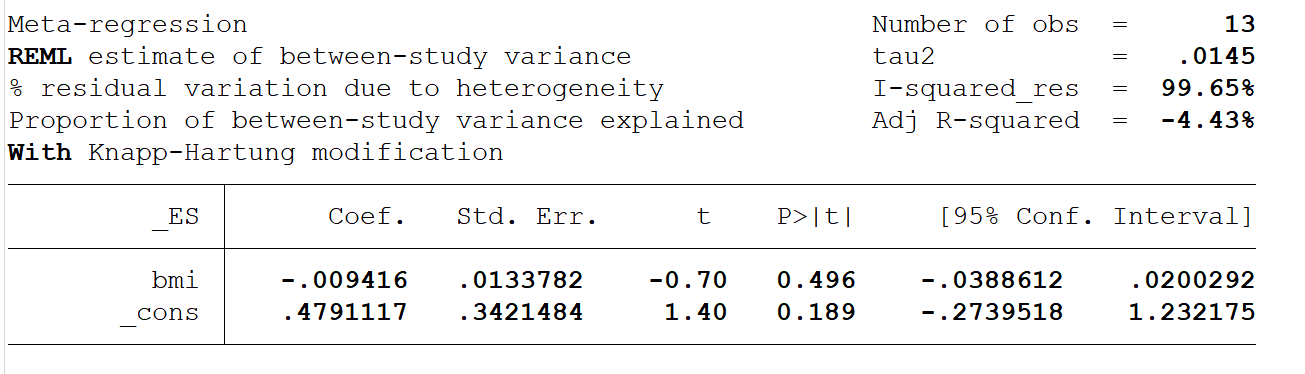


1. By the proportion of non-smokers


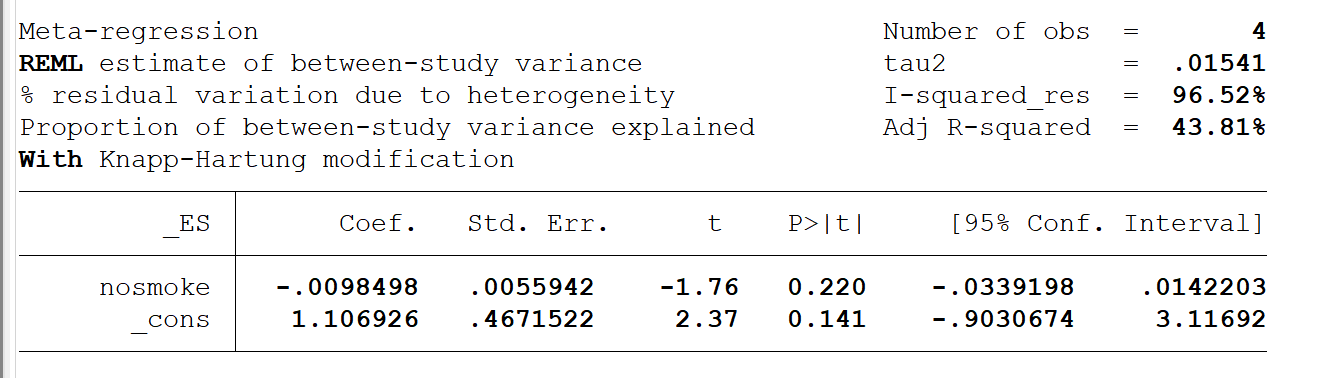


1. By systolic blood pressure
2.
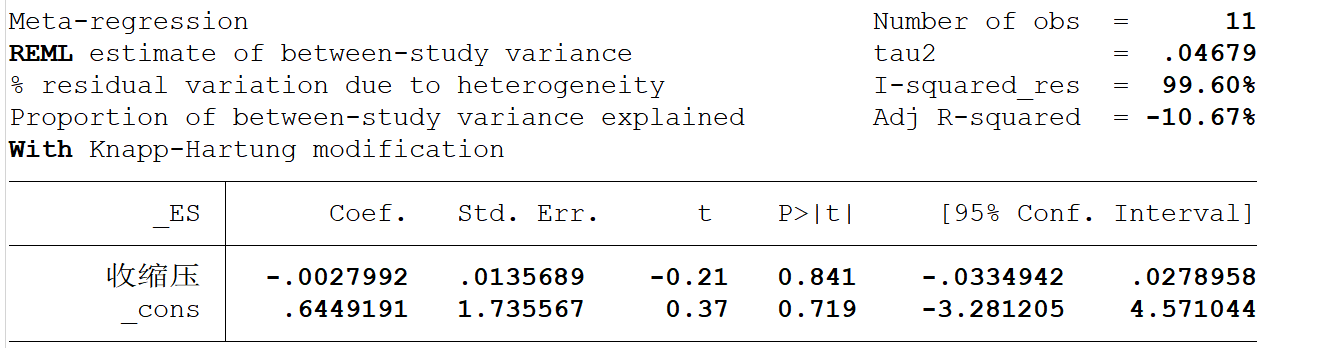
By diastolic blood pressure
3.
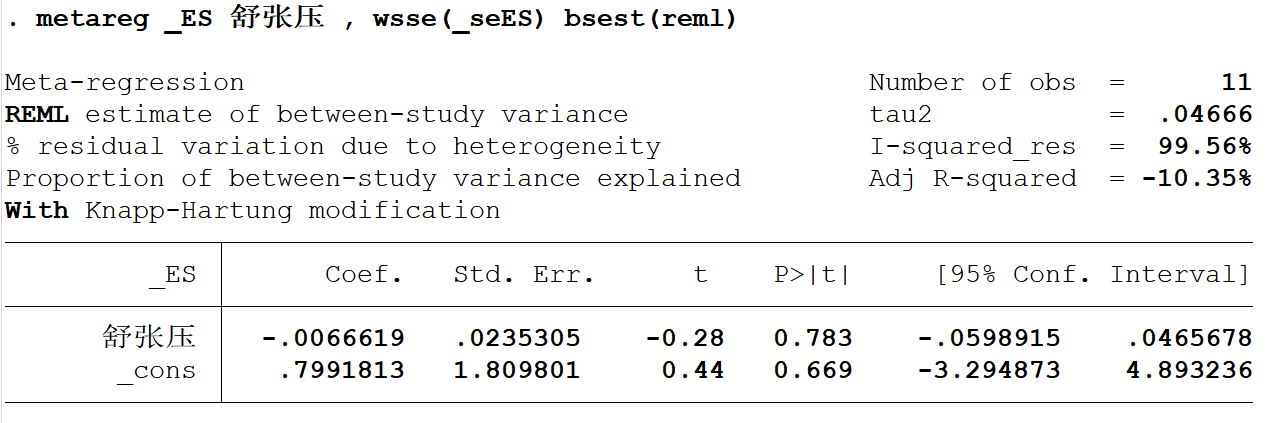
By waistline
4.
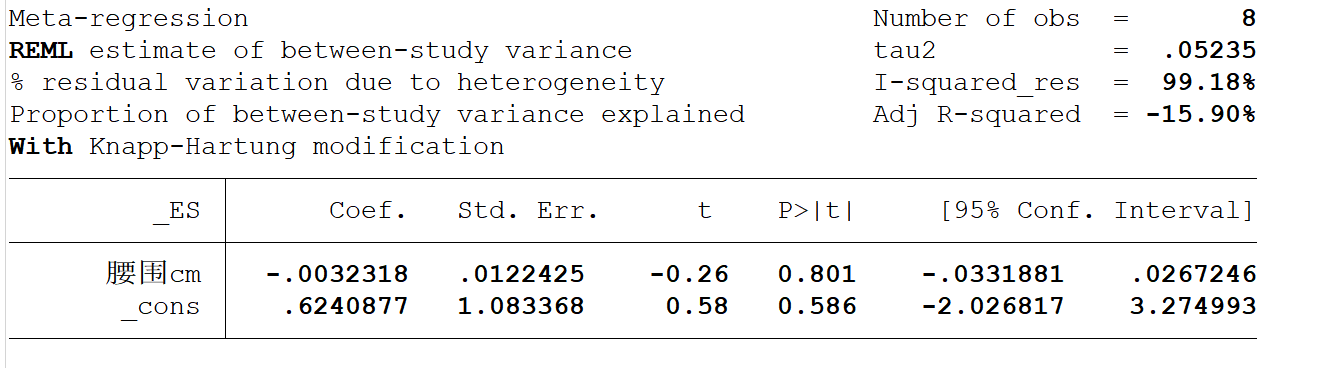
By fasting blood glucose


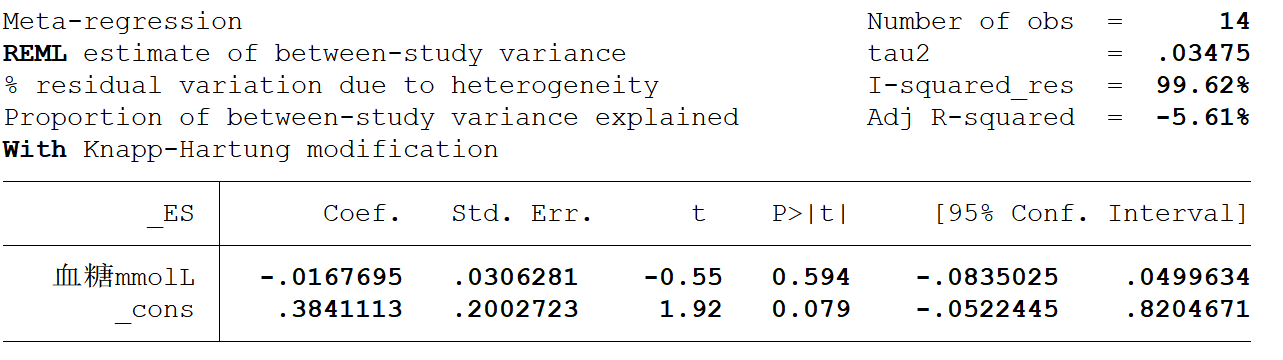


1. By ALT


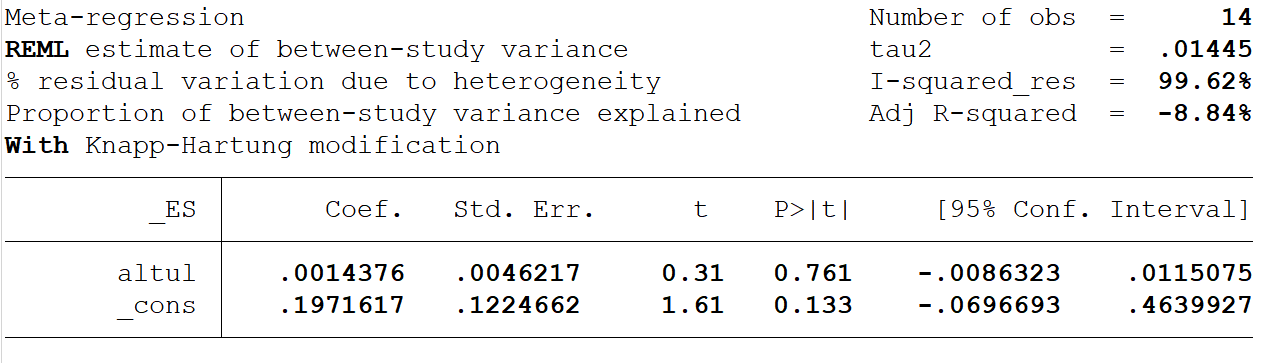


1. By AST


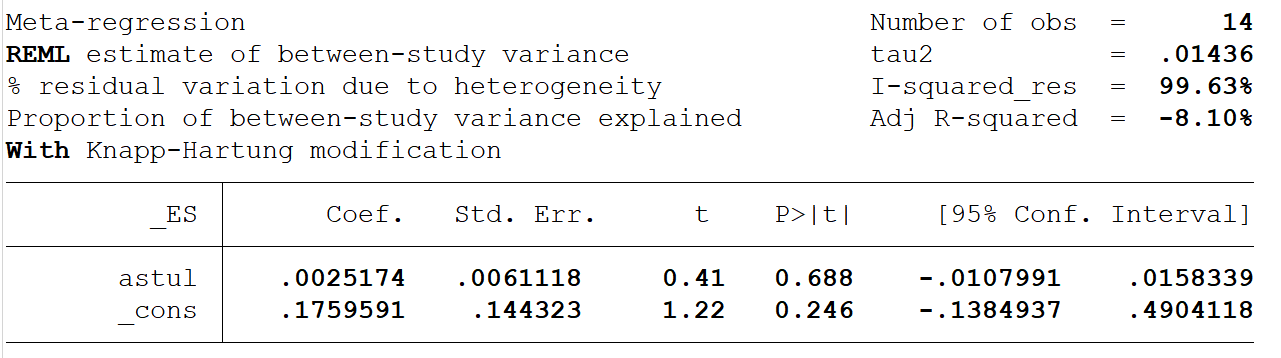


1.
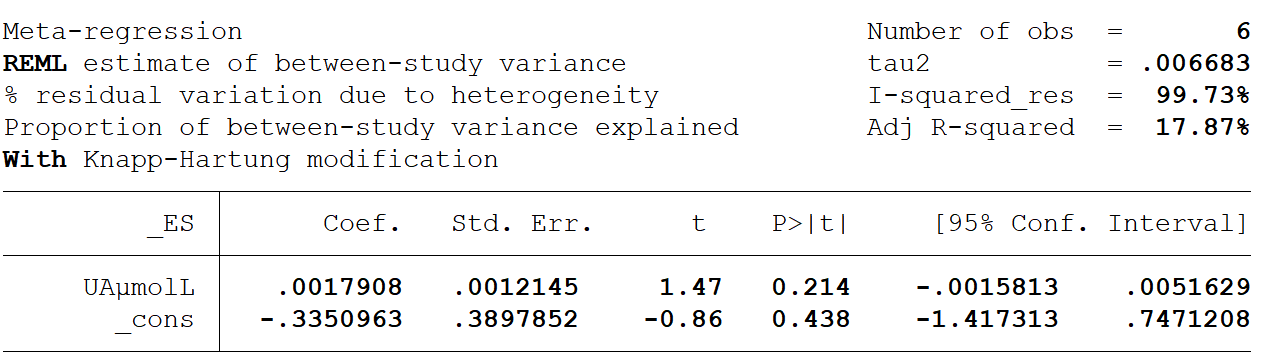
By Uric Acid

**Figure S11** Meta-regression of the Difference in AIP levels between groups (WMD)

1. By age


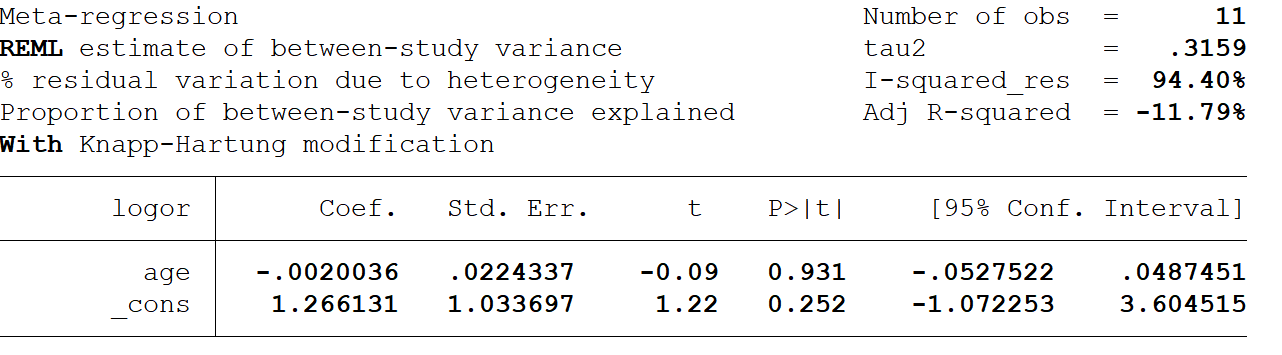


1. By Sex ratio


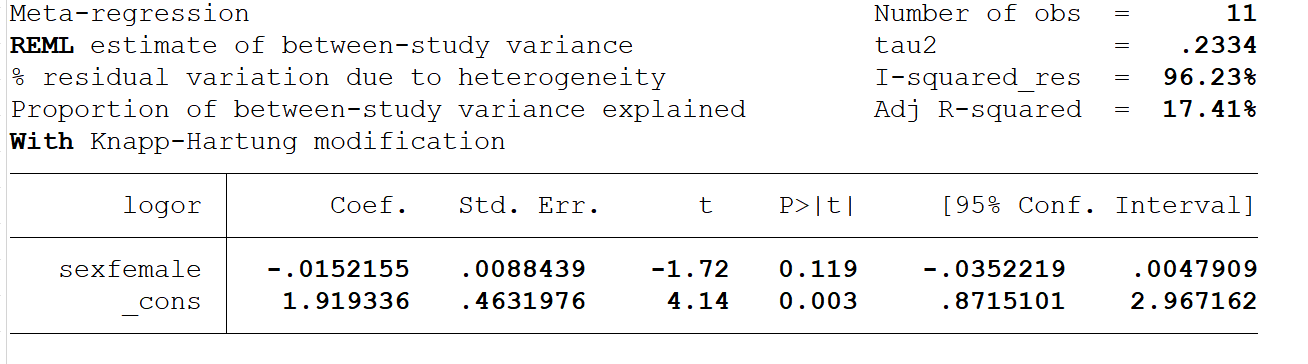


1. By BMI


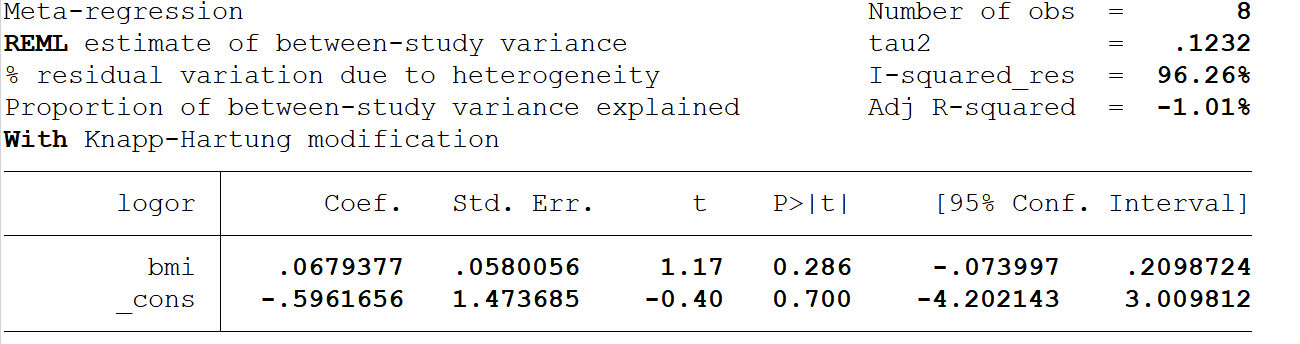


1. By the proportion of non-smokers


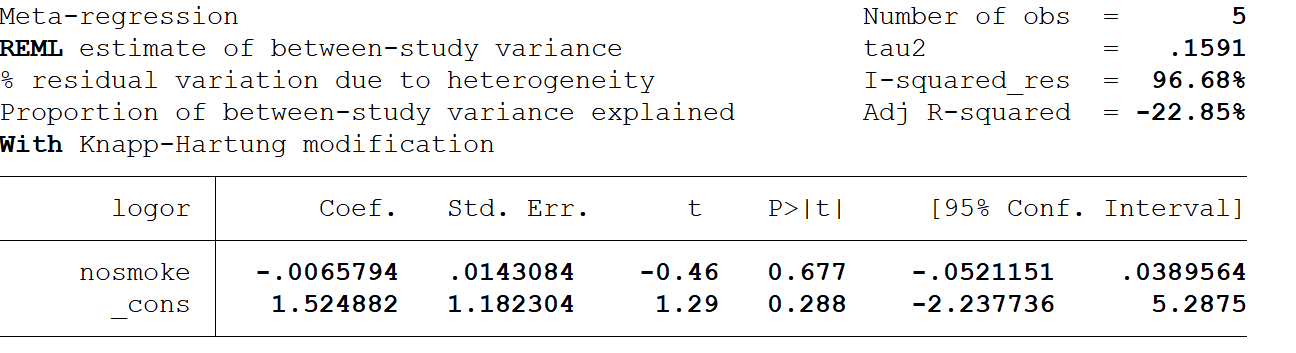


1. By systolic blood pressure


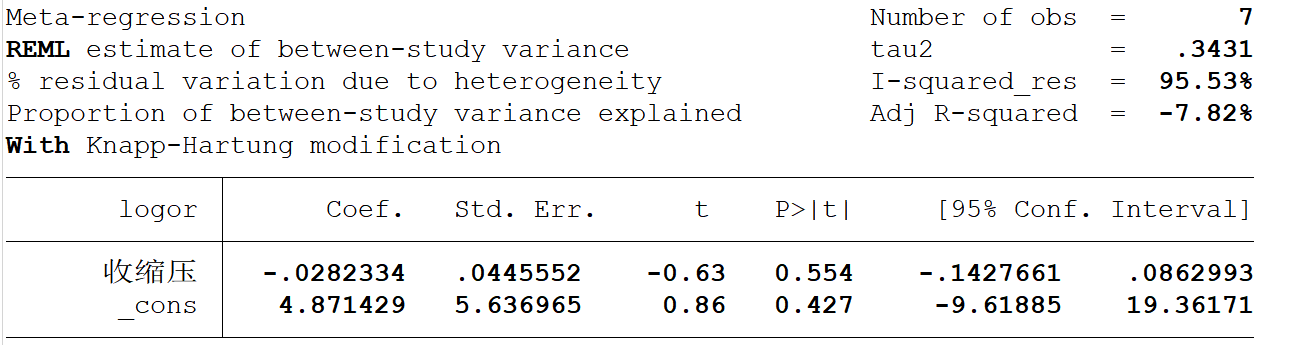


1. By diastolic blood pressure
2.
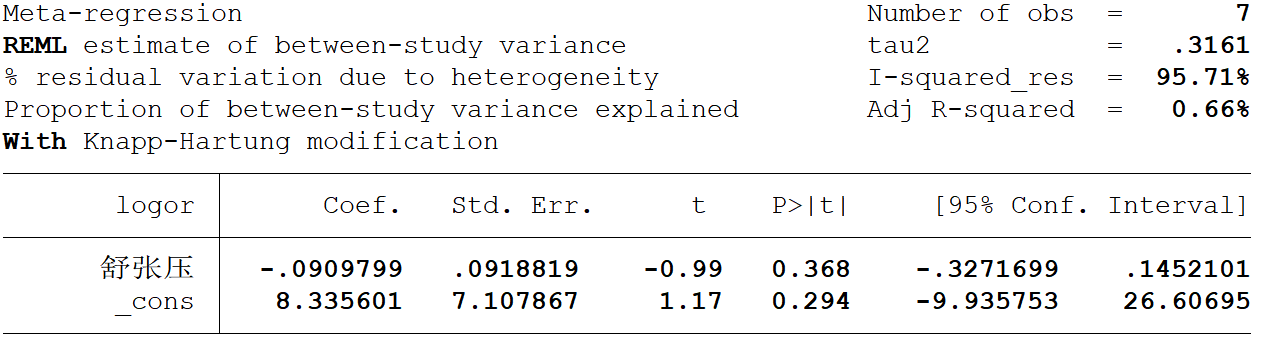
By waistline


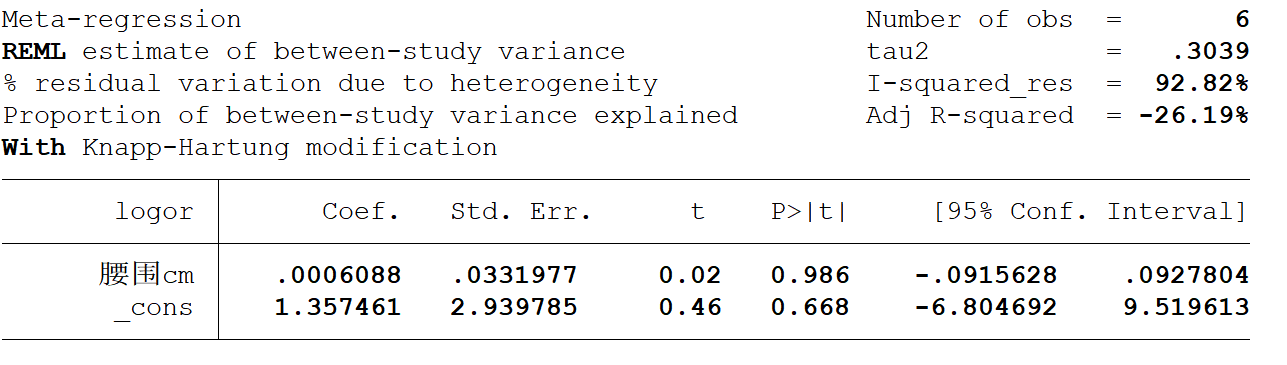


1. By fasting blood glucose


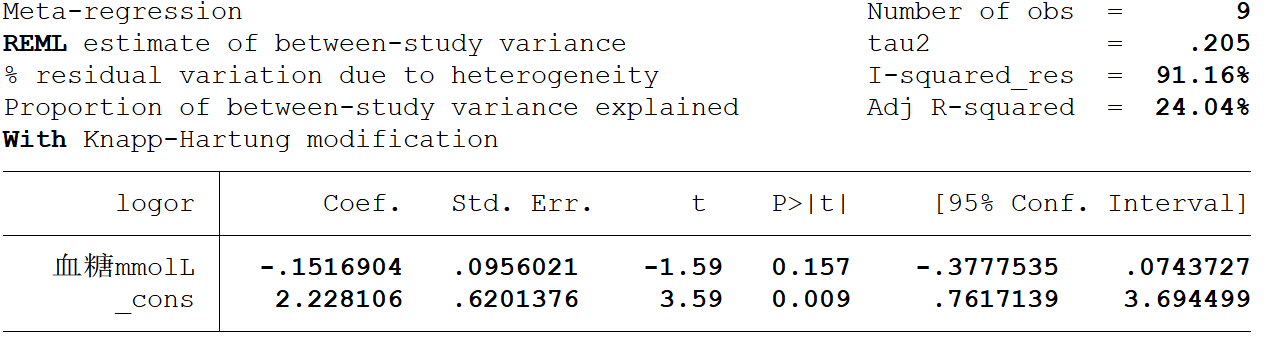


1. By ALT


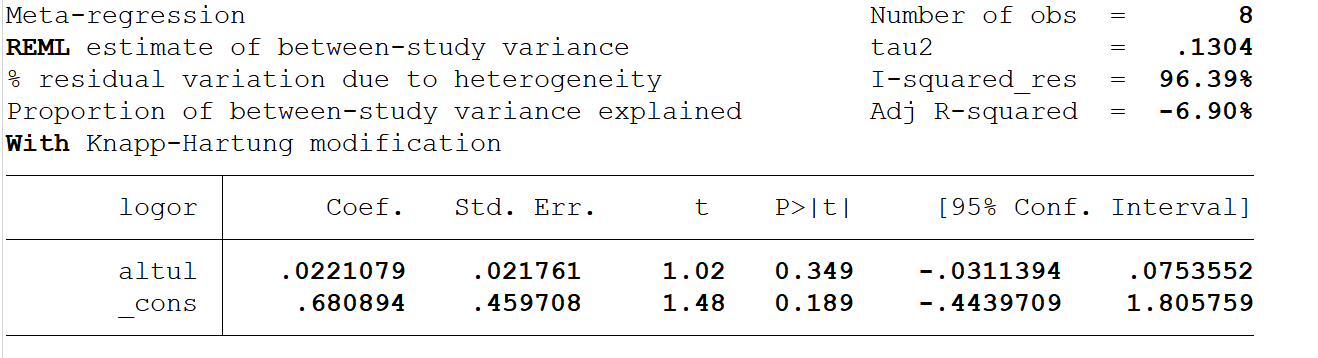


1. By AST


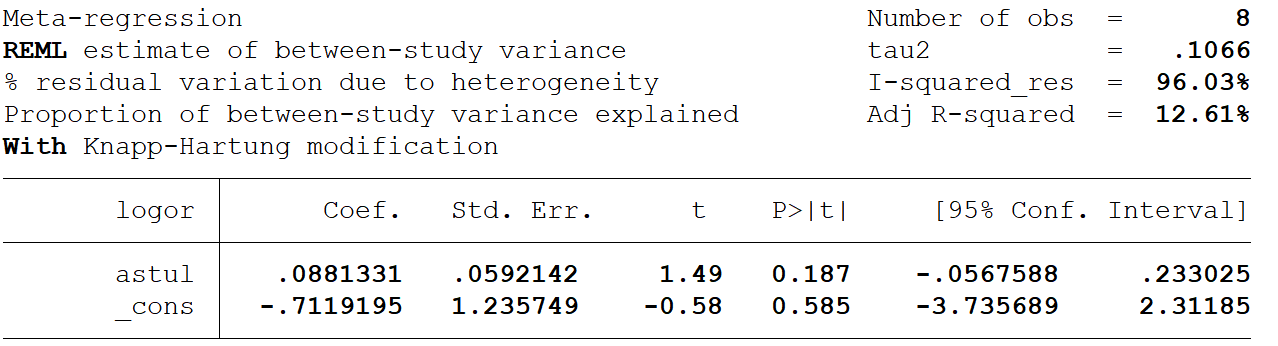


1. By Uric Acid


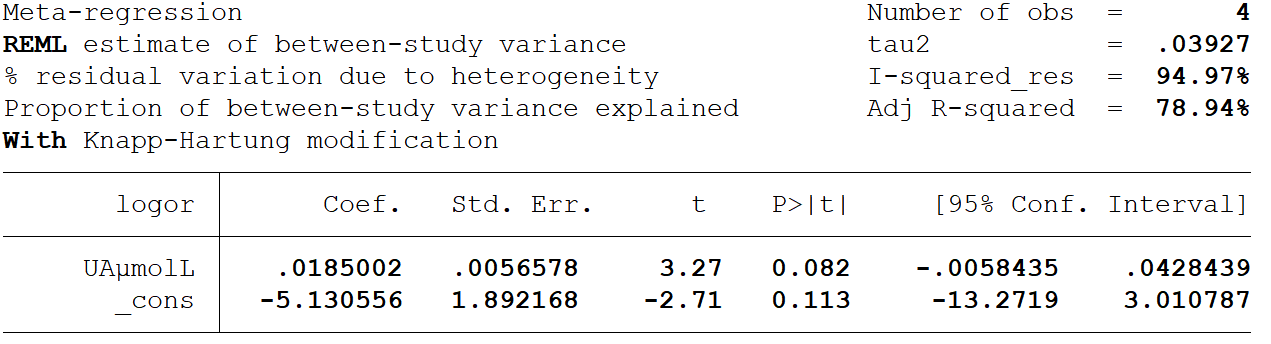


**Figure S12** Meta-regression of the association between AIP and NAFLD/MAFLD (OR)


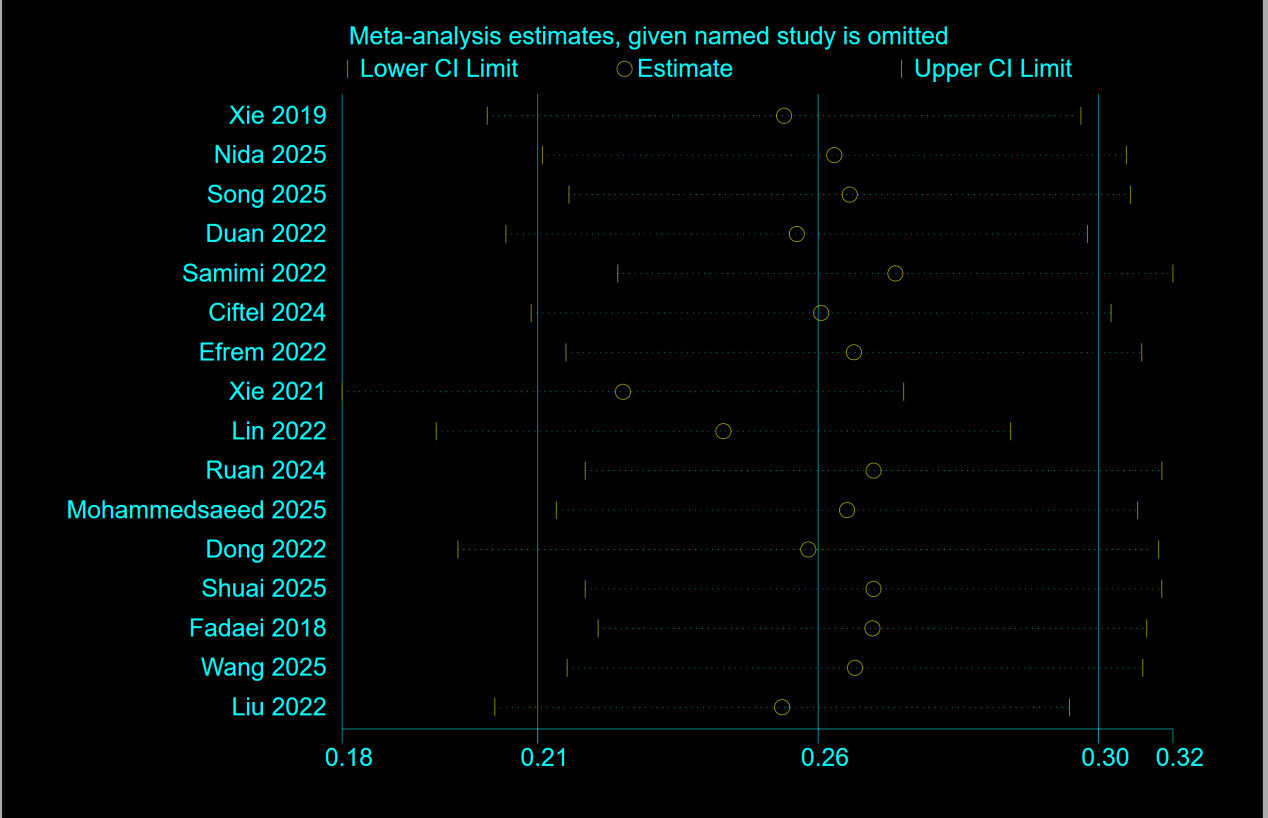


**Figure S13** Sensitivity analysis of the difference in AIP levels between groups (WMD)


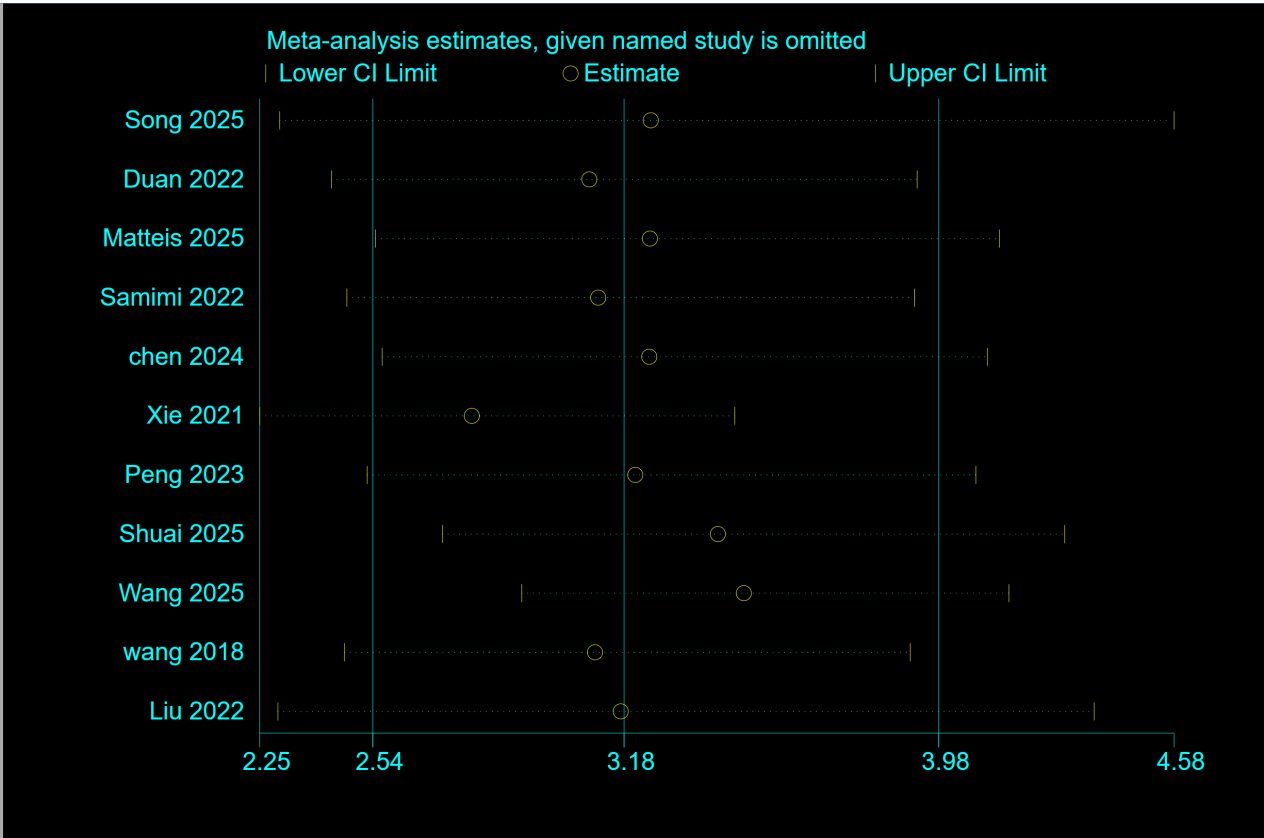


**Figure S14** Sensitivity analysis of the association between AIP and NAFLD/MAFLD (OR)


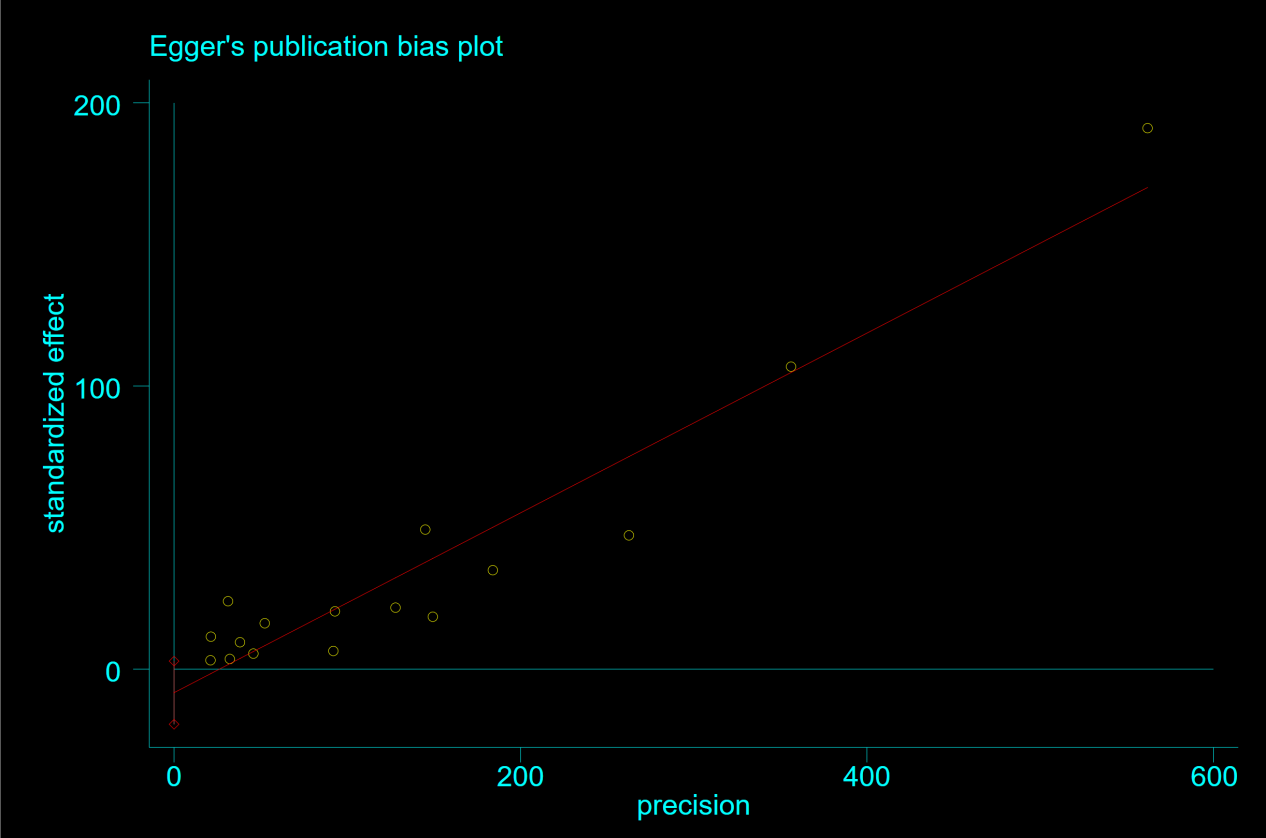


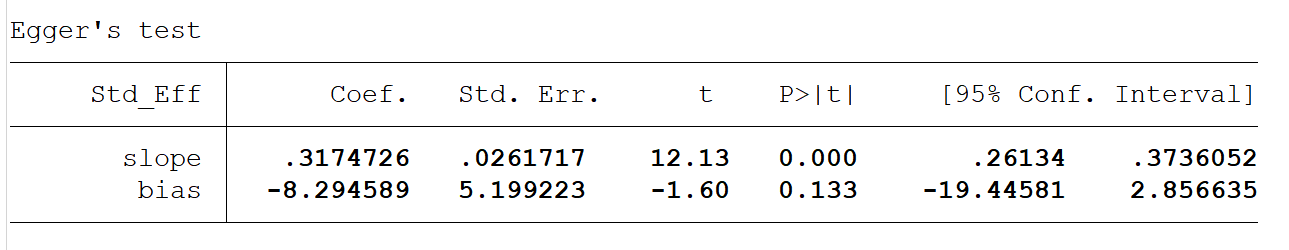


**Figure S15** Publication bias of the difference in AIP levels between groups (WMD)


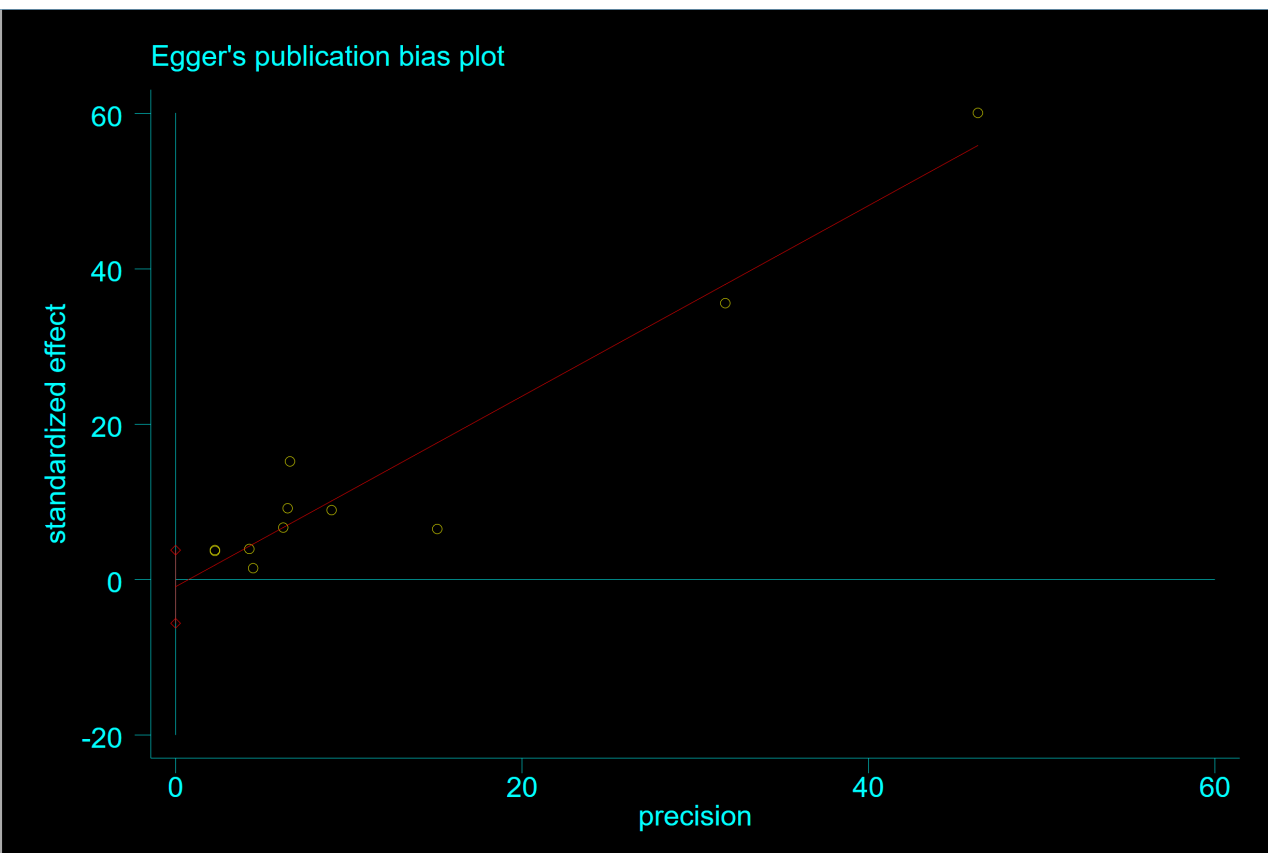


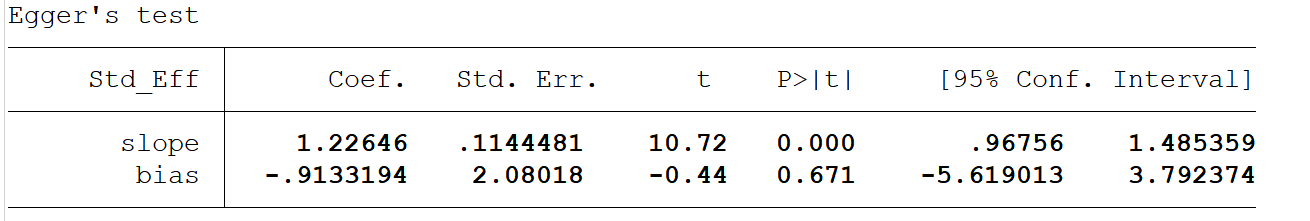


**Figure S16** Publication bias of the association between AIP and NAFLD/MAFLD (OR)


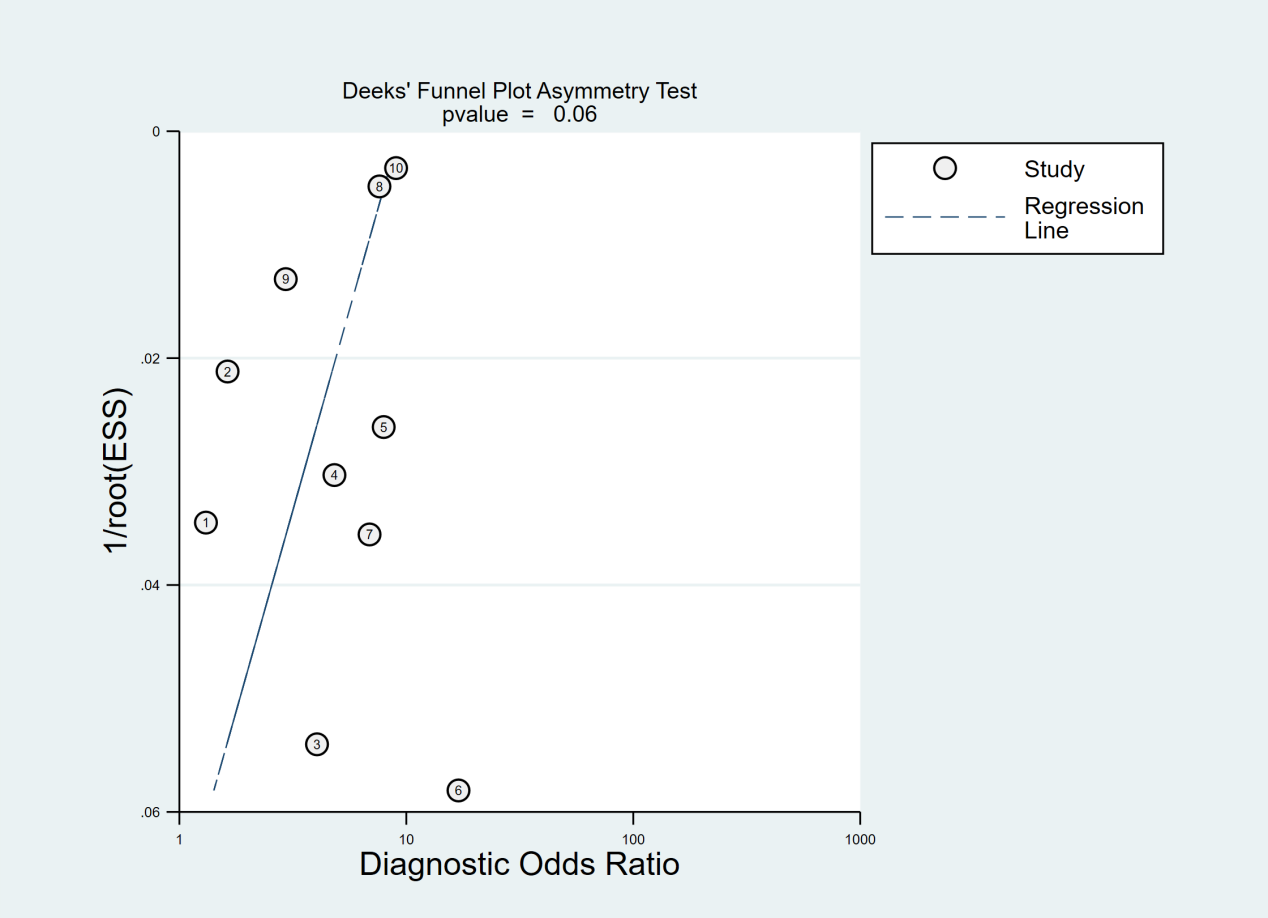


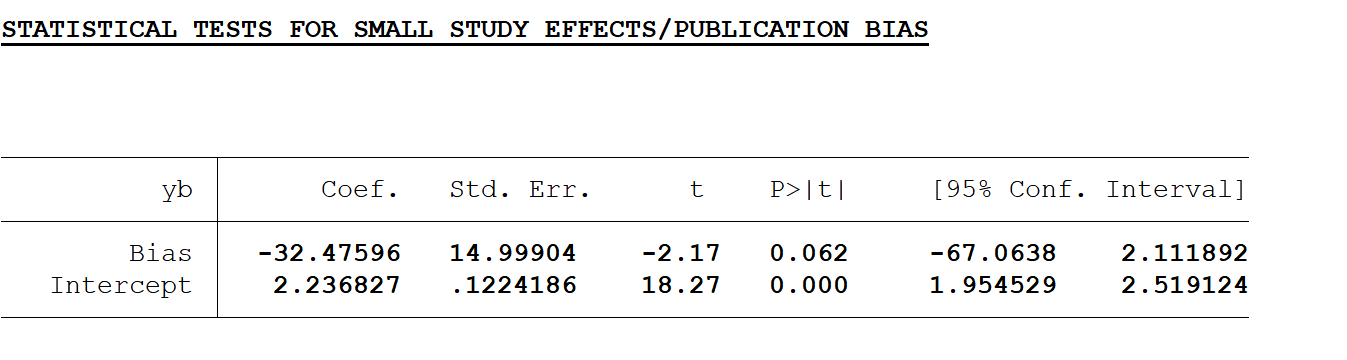


**Figure S17** Publication bias of the diagnostic performance of AIP (AUC)


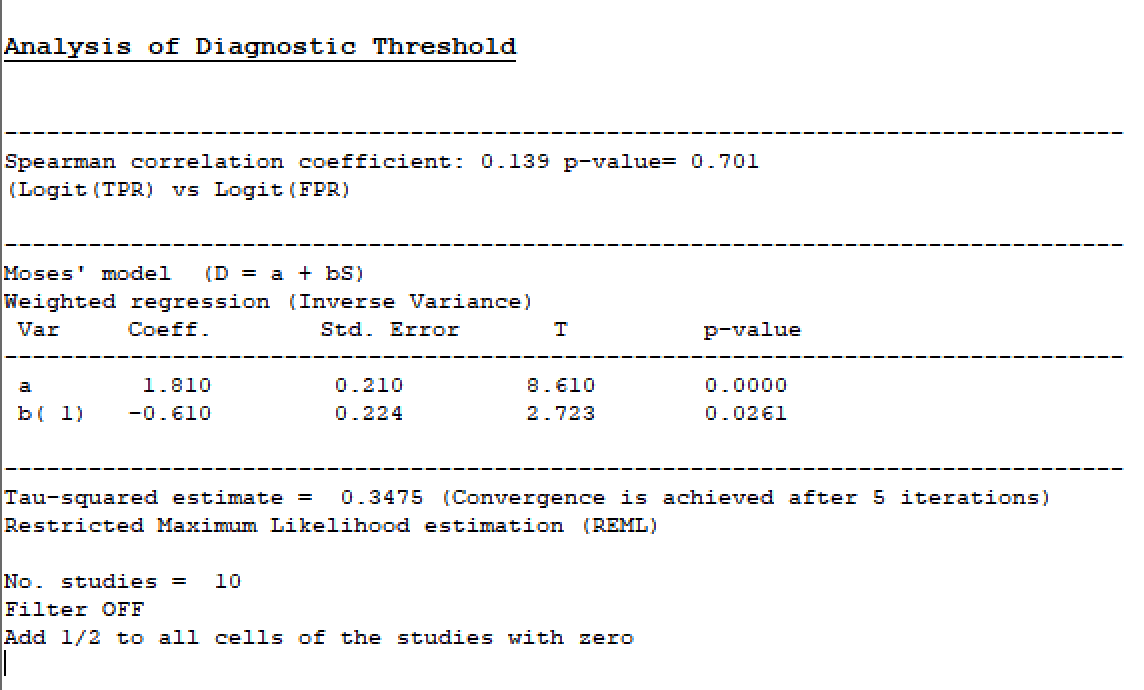


**Figure S18** Threshold effect analysis for diagnostic accuracy studies
